# Types, reporting and acceptability of community-based interventions for stillbirth prevention in sub-Saharan Africa (SSA): a systematic review

**DOI:** 10.1016/j.eclinm.2023.102133

**Published:** 2023-08-03

**Authors:** Uchenna Gwacham-Anisiobi, Yebeen Ysabelle Boo, Adetola Oladimeji, Jennifer J. Kurinczuk, Nia Roberts, Charles Opondo, Manisha Nair

**Affiliations:** a National Perinatal Epidemiology Unit, Nuffield Department of Population Health, University of Oxford, Oxford, United Kingdom; b Solina Center for International Development and Research, Nigeria; c Bodleian Health Care Libraries, University of Oxford, Oxford, United Kingdom; d London Department of Medical Statistics, London School of Hygiene and Tropical Medicine, London, United Kingdom

**Keywords:** Stillbirth, Community intervention, Foetal death, Sub Saharan Africa, Stillbirth prevention

## Abstract

**Background:**

Community-based interventions are increasingly being implemented in Sub-Saharan Africa (SSA) for stillbirth prevention, but the nature of these interventions, their reporting and acceptability are poorly assessed. In addition to understanding their effectiveness, complete reporting of the methods, results and intervention acceptability is essential as it could potentially reduce research waste from replication of inadequately implemented and unacceptable interventions. We conducted a systematic review to investigate these aspects of community-based interventions for preventing stillbirths in SSA.

**Methods:**

In this systematic review, eight databases (MEDLINE(OvidSP), Embase (OvidSP), Cochrane Central Register of Controlled Trials, Global Health, Science Citation Index and Social Science Citation index (Web of Science Core Collection), CINAHL (EBSCOhost) and Global Index Medicus) and four grey literature sources were searched from January 1, 2000 to July 7, 2023 for relevant quantitative and qualitative studies from SSA (PROSPERO-CRD42021296623). Following deduplication, abstract screening and full-text review, studies were included if the interventions were community-based with or without a health facility component. The main outcomes were types of community-based interventions, completeness of intervention reporting using the TIDier (Template for Intervention Description and replication) checklist, and themes related to intervention acceptability identified using a theoretical framework. Study quality was assessed using the Cochrane risk of bias and National Heart, Lung and Blood Institute's tools.

**Findings:**

Thirty-nine reports from thirty-four studies conducted in 18 SSA countries were eligible for inclusion. Four types of interventions were identified: nutritional, infection prevention, access to skilled childbirth attendants and health knowledge/behaviour of women. These interventions were implemented using nine strategies: mHealth (defined as the use of mobile and wireless technologies to support the achievement of health objectives), women’s groups, community midwifery, home visits, mass media sensitisation, traditional birth attendant and community volunteer training, community mobilisation and transport vouchers. The completeness of reporting using the TIDier checklist varied across studies with a very low proportion of the included studies reporting the intervention intensity, dosing, tailoring and modification. The quality of the included studies were graded as poor (n = 6), fair (n = 14) and good (n = 18). Though interventions were acceptable, only 4 (out of 7) studies explored women’s perceptions, mostly focusing on perceived intervention effects and how they felt, omitting key constructs like ethicality, opportunity cost and burden of participation.

**Interpretation:**

Different community-based interventions have been tried and evaluated for stillbirth prevention in SSA. The reproducibility and implementation scale-up of these interventions may be limited by incomplete intervention descriptions in the published literature. To strengthen impact, it is crucial to holistically explore the acceptability of these interventions among women and their families.

**Funding:**

Clarendon/Balliol/NDPH DPhil scholarship for UGA. MN is funded by a 10.13039/501100000265Medical Research Council Transition Support Award (MR/W029294/1).

**Research in context**

**Evidence before this study**

Cochrane systematic reviews have previously synthesised the effectiveness of community-based interventions in improving maternal and newborn outcomes and reducing stillbirths, but none has systematically assessed the completeness of reporting of the intervention, and its acceptability among women and other stakeholders with a focus on sub-Saharan Africa. A systematic review of community-based interventions was conducted including published and unpublished literature between January 1, 2020 and July 7, 2023 and the following search terms were used: “community-based interventions”, “stillbirths”, “perinatal death”, “sub-Saharan Africa”, “pregnancy”, “birth attendants”, “home visits”, “women’s groups”, “mobile health"," health promotion”, “nutritional interventions”, “smoking cessation”. Thirty-nine reports from thirty-four studies conducted in eighteen SSA countries were eligible for inclusion with interventions targeting nutrition, infection prevention, access to skilled childbirth attendants and health knowledge/behaviour of women. The completeness of reporting using the TIDier checklist varied across studies and 18 of 34 studies were classified as good quality. In terms of acceptability, only 4 studies explored women’s perceptions, but key constructs like ethicality, opportunity cost and burden of participation were omitted.

**Added value of this study**

This review emphasises the importance of comprehensive evaluations of community-based interventions, including their acceptability, and the need for better reporting. Measuring effectiveness alone is insufficient.

**Implications of all the available evidence**

The increasing implementation and evaluation of community-based interventions for stillbirth prevention in SSA presents a timely opportunity to optimise investments, but they need to be appropriately documented to facilitate replication and implementation scale-up. The acceptability of interventions to all stakeholders is crucial, particularly women. Vital aspects of intervention acceptability like the ethicality, burden of participation and opportunity cost for women should be evaluated and documented.

## Introduction

Globally, in every 16 seconds of 2019, one baby was born without signs of life (i.e. stillborn) at 28 weeks of pregnancy or later.^1^ About 98% of the estimated two million global stillbirths in 2019 occurred in low- and middle-income countries (LMICs); 44% of these were in sub-Saharan Africa (SSA), with Nigeria bearing most of the burden.^2^ Stillbirths can occur before the onset of labour (antepartum) or during labour and childbirth (intrapartum). It is estimated that over 40% of global stillbirths occur in the intrapartum period.^3^ In SSA, about 50% of all stillbirths are intrapartum^2^ while the others occur antepartum when women are likely still within their communities. Despite decades of intervention for maternal, perinatal and newborn health in SSA, little progress has been made as the region continues to record very high rates of stillbirths as well as maternal and newborn deaths.^4^

Community-based interventions for stillbirth prevention have received increased policy attention in the past two decades for SSA and other regions with a large burden of stillbirths.^5^^,^^6^ Lessons shared at the 53rd safe motherhood and emergency obstetrics WHO regional committee for Africa meeting in August 2003 in South Africa and the joint International Conference on community health convened in October 2006 in Ethiopia^5^ called for stronger community involvement in planning, implementation and monitoring interventions to improve maternal and newborn health outcomes including stillbirths. Several community-based interventions have been implemented in SSA, but their reporting and acceptability to women and health workers have been poorly studied.^7^ Understanding the types, completeness of reporting and acceptability of the interventions is equally important as measures of the effects of these interventions. This information would enable both researchers and policymakers to improve the replicability and implementation of the tested community-based interventions and optimise benefits. Incomplete description of the intervention and lack of acceptability assessment could lead to lower trust in the reported effectiveness of interventions. A pluralistic approach to intervention assessment which looks at the efficacy (to what extent does the intervention produce the intended outcomes in experimental or ideal settings), effectiveness (to what extent does the intervention produce the intended outcomes in real world settings), theory based (what works in which circumstances and how), and systems (how do the system and intervention adapt to one another) has been recommended for complex interventions.^8^ However, the usefulness of the insights drawn from such assessments hinges on the complete reporting of interventions.

There have been three previous systematic reviews of community-based interventions for maternal and newborn health, two focusing globally^9^^,^^10^ and one on Africa.^11^ Only one of these reviews focused specifically on interventions to prevent stillbirth, but this review was more than a decade ago and covered both health facility and community-based interventions worldwide.^9^ These reviews also did not systematically assess the completeness of the intervention description and did not explore the acceptability of the interventions among women and healthcare providers. Therefore, the objectives of this review were:

1. To describe the types of community-based interventions for preventing stillbirths in SSA and the strategies used to implement them.
2. To examine the completeness of reporting of community-based stillbirth prevention interventions in SSA.
3. To understand the acceptability of the interventions among health workers, women and their families, and communities.

## Methods

### Search strategy and selection criteria

A systematic review was conducted and has been reported following the PRISMA reporting guideline (see Supplementary material) and steps outlined in the study protocol registered with the International Prospective Register of Systematic Reviews (registration number CRD42021296623).^12^ Eight databases (MEDLINE(OvidSP)[1946-present], Embase (OvidSP)[1974-present], Cochrane Central Register of Controlled Trials (Cochrane Library, Wiley)[1ssue 7 of 12, July 2022], Global Health (OvidSP)[1973–2022 week 28], Science Citation Index and Social Science Citation index (Web of Science Core Collection)[1900-present], CINAHL (EBSCOhost)[1982-present] and Global Index Medicus https://www.globalindexmedicus.net/) and four grey literature sources (ProQuest Dissertations and Theses - Global, www.who.int/trialsearch/, www.ClinicalTrials.gov and Google (conference proceedings, and implementation reports) were searched from January 1, 2000 to July 7, 2023, for relevant quantitative and qualitative studies.

The search terms used were synonyms and specific terms from different types of related interventions implemented in the community in similar reviews found during the scoping search. They included “community-based interventions”, “stillbirths”, “perinatal death”, “sub-Saharan Africa”, “pregnancy”, “birth attendants”, “home visits”, “women’s groups”, “mobile health"," health promotion”, “nutritional interventions”, “smoking cessation”. UGA developed the initial search strategy, which was reviewed by MN and finalised by the University Librarian NR. The search terms and search strings were customised to each database, register and search engine. Boolean operators “OR” between synonyms and “AND” between search strings were applied to widen the scope of the search without losing its focus.^13^ The search terms were piloted on MEDLINE, the inclusion and exclusion criteria were applied to five percent of the papers retrieved to check the reliability for identifying relevant papers. The full search strategy is shown in the Supplementary material. The search was first conducted on December 16, 2021 by UGA and reviewed by NR. An updated search was conducted on July 7, 2023.

### Inclusion and exclusion criteria

Studies were included if the interventions targeted stillbirth reduction exclusively or as part of a complex intervention in SSA (SSA is defined geographically as all African countries excluding the five countries of northern Africa). There were no restrictions based on study design or language. Included studies were community-based with or without a health facility component. Studies were excluded if they were social and economic interventions having indirect impact on stillbirths such as micro credit women empowerment schemes, however, studies which provided vouchers for women to ease transportation to health facilities were included. Hospital-only interventions and maternal waiting home interventions were excluded as women in waiting homes had daily access to health workers^14^ and were not in their homes.

### Study selection

Literature screening and study selection were undertaken independently by three reviewers. UGA conducted independent title and abstract screening of all retrievals while YYB and AO served as second assessors screening 50% each of all titles and abstracts. Next, all studies included for full text screening were screened by UGA, while YYB and AO screened 40% of these studies (20% each) to compare with UGA’s decisions to keep or remove the studies. Disagreements at both stages were resolved by consensus and by referring to the review protocol. Disagreements which were unresolved were further discussed with MN. A decision history record was maintained throughout the process.

### Quality assessment and robustness of reporting

UGA, YYB and AO independently assessed study quality using the Cochrane risk of bias tool, National Heart, Lung and Blood Institute's tools and the Critical Appraisal Skills Programme (CASP) checklist. The overall quality rating for cluster randomised controlled trials (cRCTs) and RCTs was based on the assessment rating of all five domains of the Cochrane risk of bias tool. For the pre-post design studies, a score of 1 was given to each of the questions on the tool where the characteristic assessed was present in the paper being assessed. Papers scoring 70% of the available scores and above were rated as good quality, while papers that scored 50%–69% and 0%–49% were rated as fair and poor quality, respectively. Similar scoring and rating techniques were utilised for cross-sectional, cohort and qualitative studies.

### Data extraction and analysis

A data extraction tool was designed using Microsoft Excel to capture information about study design, intervention characteristics and other contextual information related to the review questions. None of the authors of included papers was contacted for additional information, as the review team believed they had sufficient information to conduct the analysis. The characteristics of the studies included were described using a summary table. Referring to the registered protocol, the main outcomes of this review were types of community-based interventions, themes related to intervention acceptability identified using a theoretical framework and intervention effect on stillbirths. However, due to the observed variability in completeness of reporting, completeness of intervention reporting using the outcome (Template for Intervention Description and replication) checklist was added as an outcome. Three outcomes of the review, intervention types, reporting, and acceptability, are presented in this paper, and the fourth outcome, effectiveness, will be presented as a meta-analysis in a separate follow-on paper.

UGA extracted data from 100% of the included papers, and compared data with YYB and AO who independently extracted data from separate 50% of the included studies. Key information extracted from identified interventions were: what the study/project sought to achieve (types of intervention)^9^ and the modality of intervention implementation (intervention delivery strategy).^10^ The types of interventions and delivery strategy were grouped and then described using a narrative synthesis. The TIDier checklist^15^ was used to assess the completeness of reporting for the interventions. This tool was developed by Hoffmann et al., to aid researchers, clinicians and patients to reliably report, implement, replicate and build on complex interventions.^15^ Sections of the checklist which were reported fully in papers were marked as ‘reported’ and the sections missed were marked ‘not reported’ on the extraction sheet. The frequency of reporting for each section/domain was calculated and described using a narrative synthesis.

Acceptability was defined as “a multi-faceted construct that reflects the extent to which people delivering or receiving a healthcare intervention consider it to be appropriate, based on anticipated or experienced cognitive and emotional responses to the intervention”.^16^ The questions underpinning the Sekhon et al., acceptability framework^16^ served as a guide for data extraction for acceptability of interventions; both description and supporting quotes related to the seven component constructs, shown in Panel 1, were extracted. If data for any section of the framework was not available in a given paper, it was noted as ‘not reported’ on the extraction sheet. A framework analysis using the acceptability framework was conducted,^17^ which entailed five stages—familiarisation with data, identification of themes, indexing, charting and mapping/interpretation. Familiarisation with the data involved reading the secondary data in the included papers to get a holistic sense of what researchers have found in the included studies. Thereafter, rather than identifying themes and building a framework, the seven component constructs of the framework (see Panel 1)^16^ were used as deductive themes for the analysis.

*Panel 1* **Intervention Acceptability framework (Sekhon et al.,16).**

Acceptability framework.

1. **Affective attitude:** refers to how an individual feels about the intervention
2. **Burden:** The perceived amount of effort that is required to participate in the intervention
3. **Ethicality:** The extent to which the intervention fit with an individual’s value system
4. **Intervention coherence:** the extent to which the participant understands the intervention and how it works.
5. **Opportunity costs:** the extent to which benefits, profits or values must be given up to engage in the intervention
6. **Perceived effectiveness:** The extent to which the intervention is perceived as likely to achieve its purpose
7. **Self-efficacy:** the participant’s confidence that they can perform the behaviour(s) required to participate in the intervention.

Two reviewers (UGA and AO) independently coded the extracted data and met to agree on suitable subthemes for each construct/theme. The data from the included papers were then independently indexed by carefully reading included quotes in each paper and systematically applying them to the appropriate component construct of the framework. UGA and AO discussed and resolved any disparities in how data were indexed. Charting the data involved both researchers organising the indexed data into a manageable format using matrix table showing the construct/theme, subtheme and the supporting quotes. The final stage was mapping and interpretation, which involved finding patterns and making sense of the data while reflecting on the review question. This was done by reflexive conversations involving UGA, AO and MN. The charted data were reviewed by UGA, AO, MN, CO and JK and utilised reflexive conversations to interpret the data. A narrative synthesis was utilized to consolidate the findings from the qualitative analysis across the different themes.

### Ethical statement

Ethical approval was not applicable for this study, as this was a systematic review of existing literature and did not involve direct contact with human subjects.

### Role of the funding source

The study funders had no role in the design, data collection, analysis, or interpretation of findings. UGA, YYB, AO, NR, JJK, CO and MN had access to the data in the study and accept responsibility for the decision to submit for publication.

## Results

**Fig. 1:**
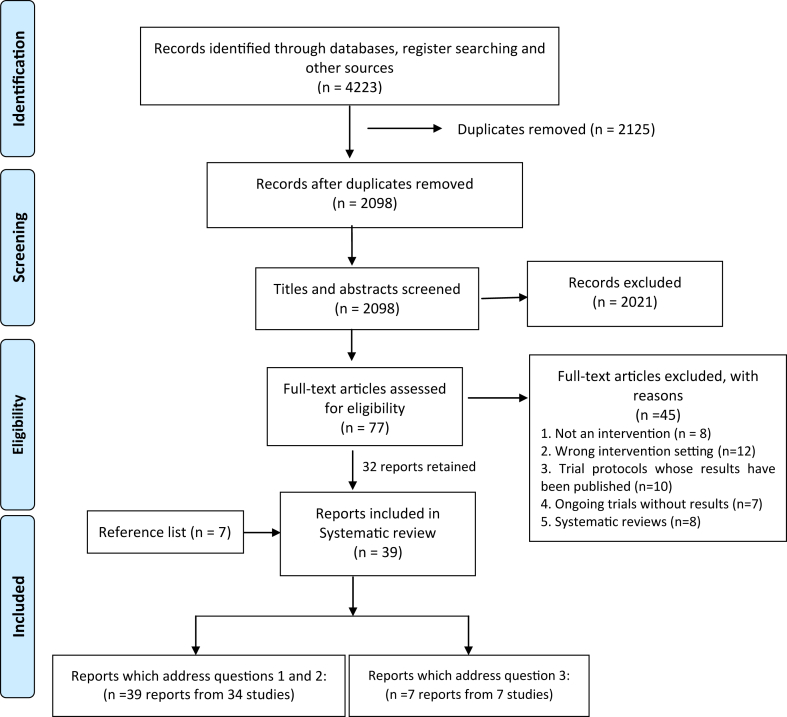
**The PRISMA chart showing the systematic search and inclusion of studies**.

The searches yielded a total of 4223 records (Fig. 1). Following deduplication, the title and abstracts of 2098 records were screened independently by three reviewers and 77 were retained for full-text review. Forty-five records were excluded based on the inclusion/exclusion criteria and seven further records were included after reference list searches. All 39 papers/reports included addressed objectives one and two while seven addressed objective three.

When there was more than one report retrieved for a study, the records were merged and reported as one study. In situations where these reports described different aspects of the one study; for example, intervention description and user experience; both records were utilised, but they contributed data for two separate review objectives.

**Fig. 2:**
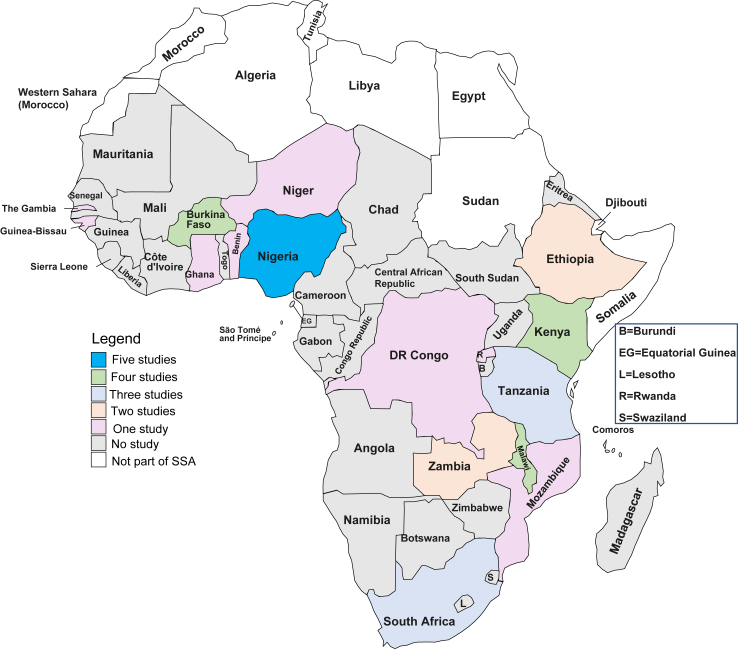
**Geographical distribution and frequency of included studies.** The map in Fig. 2 displays all the countries in Africa and the number of studies included from each country in SSA. The legend indicates that countries coded blue have 5 studies included, green has 4 studies, powder blue has 3 studies, light orange has 2 studies, liliac has 1 study, grey has no study, and white is not part of SSA.

The 39 reports included in this review were from 34 studies [Table 1] conducted in 18 of the 49 countries in SSA. East Africa had the highest number of studies (sixteen from Malawi, Mozambique, Zambia, Ethiopia, Kenya, Tanzania, Rwanda and Uganda), followed by West Africa (fourteen studies from Burkina Faso, Benin, Gambia, Guinea-Bissau, Ghana, Niger and Nigeria). Southern Africa had three studies from South Africa, while Central Africa had one study from Democratic republic of Congo. The geographical spread and frequency of included studies are shown in Fig. 2.

**Table 1:** Summary characteristics of included studies.

Study ID	Name of study	First author and year	Key intervention(s)	Control	Country	Study design	Place of intervention	Research question addressed	Quality rating
01	Thesis	Mohammed 2016^18^	Early access to Iodized salt distribution	Later access to Iodized salt	Ethiopia	cRCT	Community only	1,2	Some concerns
02	Maikhanda	Colbourn 2013^21^	Participatory women groups to improve care practices and health-seeking behaviours using quality improvement methods	Usual care	Malawi	cRCT	Community and hospitals	1,2	Low risk
02b	Maikhanda	Colbourn 2013^54^	Participatory women groups to improve care practices and health-seeking behaviours using quality improvement methods	Usual care	Malawi	cRCT	Community and hospitals	1,2	Low risk
03	Maimwana	Lewycka 2013^22^	Women groups, peer health counselling, home visits	Usual care	Malawi	cRCT	Community and hospitals	1,2	Some concerns
03b	Thesis/Maimwana	Lewycka 2011^53^	Women groups	Usual care	Malawi	cRCT	Community only	1,2	Some concerns
04	Wired mothers	Lund 2014^23^	Automated and directed health messaging. Call voucher system for women to reach health workers.	Usual care	Tanzania	cRCT	Community only	1,2	Some concerns
05	Global network first breath trial	Pasha 2013^24^	Community mobilisation to establish and maintain emergency transport through loan schemes. TBA training, and referral linkages	Usual care	India Pakistan, Kenya^a^, Zambia^a^, Guatemala and Argentina	cRCT	Community and hospitals	1,2	Some concerns
06	First breathe trial	Matendo 2011^19^	Emergency newborn care training for TBAs, nurses and midwives.	Usual care	DRC	cRCT	Community only	1,2	Some concerns
07	Community scheduled Malaria screening and treatment (CSST)	Scott 2019^20^	Malaria screening and treatment, home visits.	Usual care	Gambia, Burkina Faso and Benin	cRCT	Community only	1,2	Low risk
08	N/A	Leight 2018^25^	Provision of birth kits, health education using Community resource persons.	Usual care	Nigeria	cRCT	Community only	1,2	High risk
09	N/A	Ilboudo 2022^26^	Home visits, health worker training, iron and folic acid supplementation, Intermittent Preventive malaria therapy, nutrition counselling	Usual care	Burkina Faso	cRCT	Community and hospitals	1,2	Low risk
10	N/A	Kone 2023^27^	Home visits, nutritional counselling, micronutrient supplementation.	Usual care	Côte d’Ivoire	cRCT	Community only	1,2	Some concerns
11	N/A	De Kok 2022^30^	Provision of prenatal fortified balanced energy-protein supplement, iron and folic acid supplementation	Usual care	Burkina Faso	RCT	Community only	1,2	Low risk
12	N/A	Alexander 2018^28^	Provision of clean cook ethanol stoves, home visits and health education.	Usual care	Nigeria	RCT	Community only	1,2	Low risk
13	Bandim Health project	Kaestel 2005^29^	Micronutrients supplementation and home visits.	Usual care	Guinea-Bissau	RCT	Community only	1.2	Low risk
14	TBA Manica program	Gloyd 2001^31^	Periodic TBA re/training and provision of essential supplies.	One off national TBA training	Mozambique	nRCT	Community only	1,2	Moderate risk
15	Taiwan medical mission and the TBA project	Chen 2011^33^	Periodic TBA re/training and provision of essential supplies.	One off training for TBAs	Malawi	Pre-post	Community only	1,2	Fair
16	Maternal and newborn health in Ethiopia partnership	Sibley 2014^32^	Training TBAs and other community health workers, health sensitisation and community health quality improvement.	N/A	Ethiopia	Pre-post	Community only	1,2	Good
17	Skilled care initiative	Hounton 2009^37^	Community mobilisation for health advocacy behavioural change communication and capacity strengthening.	N/A	Burkina Faso	Pre-post	Community and hospitals	1,2	poor
18	N/A	Sloan 2018^34^	Community mobilisation, TBA training, referral linkages, and supply of essential medications.	N/A	Nigeria	Pre-post	Community and hospitals	1,2	Good
19	Boresha	Shikuku 2020^36^	Community midwifery, training and deployment of health workers to hard to reach areas.	N/A	Kenya	Pre-post	Community only	1,2	Poor
20	N/A	Seim 2014^35^	Evacuation of women in obstructed labour from their home to a midwife, health centre, or hospital.	N/A	Niger	Pre-post	Community only	1,2	Fair
21	Text4Life	Okonofua 2023^38^	Text-based health education, access to health providers, emergency transport, health worker and volunteer emergency readiness training.	N/A	Nigeria	Pre-post	Community and hospitals	1,2	Good
2	Saving mothers giving life	Serbanescu 2019^55^	Health sensitisation, community outreach clinics and distribution of childbirth kits.	N/A	Uganda and Zambia	Pre-post (mixed)	Community and hospitals	1,2	Poor
22b	Saving mothers giving life	Conlon 2019^52^	Health sensitisation, community outreach clinics and distribution of childbirth kits.	N/A	Uganda and Zambia	Pre-post (mixed)	Community and hospitals	1,2	Poor
22c	Saving mothers giving life	Palaia 2019^48^	Health sensitisation, community outreach clinics and distribution of childbirth kits.	N/A	Uganda and Zambia	Qualitative (Mixed)	Community and hospitals	1,2,3	Fair
23	N/A	Azaare 2022^49^	National health policy for free maternal health care	Women accessing care prior to policy	Ghana	Qualitative (mixed)	Community only	1,2,3	Good
	N/A	Kamau 2020^45^	Iron and folic acid supplementation and home visits	N/A	Kenya	Qualitative	Community only	1,2,3	Good
25	Mom connect	Skinner 2018^44^	Text-based interventions with health promotion messages.	N/A	South Africa	Qualitative	Community only	1,2,3	Good
26	RapidSMS Rwanda	Musabyimana 2018^47^	Text-based interventions with health promotion messages. Community health workers also use the system to trigger systems for response from nearest health centre or and ambulance in emergencies.	N/A	Rwanda	Qualitative	Community only	1,2,3	Good
26b	RapidSMS Rwanda	Mwendwa 2015^56^	Text-based interventions with health promotion messages. Community health workers also use the system to trigger systems for response from nearest health centre or and ambulance in emergencies.	N/A	Rwanda	Qualitative		1,2,3	Good
27	Innovating for maternal and child health in Africa (IMCHA)	Joseph 2021^46^	Participatory women groups, community mobilisation and health education	N/A	Tanzania	Qualitative	Community only	1,2,3	Good
28	Sustainable Emergency Referral care	Patel N.D^50^	Low-cost emergency transport, health/emergency education, health worker training	N/A	Ghana	Qualitative (mixed)	Community and hospitals	1,2,3	Good
29	N/A	Fatti 2016^42^	Home visits, health education, counselling for anti-retroviral therapy initiation and adherence.	N/A	South Africa	Cohort	Community only	1,2	Fair
30	Mobile WACh NEO	Hedstrom 2022^43^	Personalised text based health education and action oriented SMS	N/A	Kenya	Cohort	Community only	1,2	Good
31	N/A	Okafor 2015^39^	TBA training and supervision	N/A	Nigeria	Cross sectional	Community only	1,2	Fair
32	National health promotion policy	Mostert 2021^41^	National health promotion policy (cigarette control), home visits and health education.	N/A	South Africa	Cross sectional	Community only	1,2	Good
33	RBF4MNH	Makuluni 2021^40^	Result based financing for stillbirth reduction.	N/A	Malawi	Cross sectional	Community and hospitals	1,2	Poor
34	M-Mama	Bryan et al. 2017^51^	Community mobilisation, emergency transport and health worker training.	N/A	Tanzania	Not reported	Community only	1,2	N/A

cRCT – cluster randomised controlled trial; nrCT – non-randomised controlled trial; RCT – randomised controlled trial; N/A – not applicable; TBA: Traditional birth attendant.

a Africa based studies.

Ten studies were cRCTs,^18^, ^19^, ^20^, ^21^, ^22^, ^23^, ^24^, ^25^, ^26^, ^27^ three were RCTs^28^, ^29^, ^30^ and one was a non-randomised controlled trial.^31^ Seven studies had a pre-post design,^32^, ^33^, ^34^, ^35^, ^36^, ^37^, ^38^ three were cross sectional,^39^, ^40^, ^41^ two were cohort studies,^42^^,^^43^ and four were qualitative,^44^, ^45^, ^46^, ^47^ three were mixed method studies,^48^, ^49^, ^50^ while one implementation report did provide sufficient detail on methods used^51^[Table 1].

Of the 34 studies included, 25 were conducted solely in the community,^18^, ^19^, ^20^^,^^22^^,^^23^^,^^25^^,^^27^, ^28^, ^29^, ^30^, ^31^, ^32^, ^33^^,^^35^^,^^36^^,^^39^^,^^41^, ^42^, ^43^^,^^46^^,^^47^^,^^49^^,^^51^ while nine had some element of health facility implementation including purchasing equipment, employing adjunct medical staff, and training health workers.^21^^,^^26^^,^^34^^,^^38^^,^^40^^,^^50^^,^^52^^,^^53^ Most studies were conducted in one country, while three studies were conducted in two or more countries^20^^,^^24^^,^^52^ [Table 1].

All 39 reports/papers included in the review were independently assessed for quality using the appropriate tool as per study design. Overall, 18 reports were rated as good quality/low risk of bias, while 14 and 6 were rated as fair and poor quality, respectively [Table 1]. One of the reports could not be rated as it was a project update report and did not provide sufficient information about the methodology of the study.

### Community-based interventions implemented for stillbirth prevention

All types of interventions targeting stillbirths implemented in a community setting in SSA were included. The interventions in the included studies were grouped by the overall aim of the intervention and the studies were categorised into four main groups [Table 2]:

1. Micronutrient supplementation to improve nutritional status of pregnant women. Nutritional interventions in this review assessed the impact of micronutrients supplementation such as iodine, iron and folic acid supplementation on perinatal health outcome including stillbirths.^18^^,^^26^^,^^27^^,^^29^^,^^30^^,^^45^^,^^47^
2. Screening, prevention and treatment of infections. Two studies in this category targeted Malaria and HIV infections^20^^,^^42^ which are endemic in SSA and have been linked to stillbirth occurrence.
3. Improving women’s health knowledge and health behaviours. This was the most common group of interventions with 22 studies.^21^^,^^22^^,^^24^, ^25^, ^26^, ^27^, ^28^, ^29^, ^30^^,^^32^^,^^34^^,^^35^^,^^37^^,^^38^^,^^41^^,^^43^^,^^44^^,^^46^^,^^47^^,^^51^^,^^52^ The researchers aimed to reduce stillbirths by influencing health behaviours through pregnancy health education such as recognising danger signs in pregnancy.
4. Increasing access to skilled childbirth attendants through training of community health workers, traditional birth attendants and reducing delays that women face in accessing skilled care. Eighteen studies were in this group.^19^^,^^21^^,^^23^^,^^24^^,^^31^, ^32^, ^33^, ^34^^,^^36^^,^^38^^,^^39^^,^^44^^,^^47^^,^^49^, ^50^, ^51^, ^52^

**Table 2:** Summary of included studies by type of intervention and delivery strategy.

Characteristics of interventions in included studies		No. of studies^a^
**Type of intervention**		
1	Nutritional	7
2	Prevention and management of infections (including HIV or Malaria)	2
3	Knowledge and behavioural (educating for ANC4, pollution, danger signs recognition)	22
4	Increasing access to skilled birth attendants	18
**Intervention delivery strategy**		
1	mHealth (use of mobile and wireless technologies to support the achievement of health objectives)	6
2	Women groups/peer counselling	3
3	Community Midwifery	1
4	Home visits (including education, health screening and infection treatment)	12
5	Mass media	5
6	Traditional birth attendant training	7
7	Volunteer and community health worker training	11
8	Community mobilisation (including advocacy, health sensitization, transport and emergency loan schemes).	15
9	Transport voucher/transfers	1

ANC4 – attending four or more antenatal visits in pregnancy mHealth interventions in this review included text-based educational programs and appointment reminders.

a Some studies have more than one type of intervention and may use one or more intervention delivery strategy

Based on how the researchers went about achieving their research aims, the studies were categorised into nine intervention delivery strategies [Table 2].

Most of the included studies implemented more than one type of intervention, using two or more intervention strategies. Interventions to improve health knowledge and behaviours were the most common type, while interventions targeting infection prevention were the least common. Community mobilisation activities including advocacy, health sensitisation, transport and emergency loan schemes were the most common strategy deployed, while community midwifery and transport vouchers were the least deployed strategy.

### Completeness of intervention reporting

All 34 studies addressed the questions in the TIDier checklist^15^ (Table 3) to varying degrees. Most interventions reported why they were carrying out the intervention, what the intervention was, who provided the intervention, how and where the intervention was delivered. About 74% of the included studies (n = 25) described the intervention intensity or dose. The least reported areas of the checklist were information about intervention fidelity (in 5 studies), modification (in 12 studies) and tailoring of interventions (in 9 studies).

**Table 3:** TIDier checklist assessment of included studies.

Template for intervention description and replication (TIDier) checklist for included studies	
Criteria	Number of studies (%), Total studies included = 34
**Why** Describe any rationale, theory or goal of the elements essential to the intervention	34 (100)
**What** Materials—describe physical or informational materials used in the intervention. Procedure: describe each of the procedures, activities and/or processes used in the intervention including enabling or support activities	34 (100)
**Who provided** For each category of intervention provider, describe their expertise, background and any specific training given	33 (97)
**How** Describe the modes of intervention delivery (eg face to face or by some other mechanism such as the internet or telephone). Was it delivered individually or in a group?	33 (97)
**Where** Describe the types of location where the intervention occurred, including any necessary infrastructure or relevant features	32 (94)
**When and how much** Describe the number of times the intervention was delivered and over what period of time including the number of sessions, their schedule, and their duration, intensity or dose	25 (74)
**Tailoring** If the intervention was planned to be personalised titrated or adapted, then describe what, why, when and how	9 (26)
**Modification** If the intervention was modified during the course of the study, describe the changes (what, why, when, and how).	12 (35)
**How well?** Planned: If intervention adherence or fidelity was assessed, describe how and by whom, and if any strategies were used to maintain or improve fidelity, describe them. Actual: if intervention adherence or fidelity was assessed, describe the extent to which the intervention was delivered as planned.	5 (15)

### Acceptability of community-based interventions among health workers, women, and communities

Eight studies included qualitative data, however only seven studies addressed objective three; six of them were assessed as good quality and one was fair quality (Table 4). Azaare et al.’s^49^ study was not included in the analysis because the researchers were focused on understanding the perceptions of health workers regarding the rise in stillbirths during the intervention. Six were single country studies, while one was conducted in two countries. The included studies utilised in-depth interviews or focus group discussions or both as study method. Sample sizes of the studies ranged from 19 to 93 participants. Most studies included both health workers and women and their families,^45^, ^46^, ^47^^,^^50^ while one study each had participants from funders/ministries of health,^48^ women alone^44^ and health workers.^54^

**Table 4:** Characteristics of studies included in the qualitative analysis.

First author and year	Country (ies)	Study design (s)	Number of participants	Health system actor of focus			Quality rating
				Women/families	Health workers	Funders and Ministries of health	
Palaia 2019^48^	Uganda and Zambia	Qualitative	46 IDIs	No	No	Yes	Fair
Joseph 2021^46^	Tanzania	Qualitative	86 IDIs	Yes	Yes	No	Good
Kamau 2020^45^	Kenya	Qualitative	19 IDIs	Yes	Yes	No	Good
Skinner 2018^44^	South Africa	Qualitative	32 IDIs, 7 FGDs	Yes		No	Good
Musabyimana 2018^47^	Rwanda.	Qualitative	93 (28 IDI, 10 FGD)	Yes	Yes	No	Good
Mwendwa 2015^56^	Rwanda.	Qualitative	14 FGDs (5–8 participants each)	No	Yes	No	Good
Patel et al. (No date)^50^	Ghana	Mixed	16 FGDs and 59 IDIs)	Yes	Yes	No	Good

IDIs – In-depth interviews, FGDs – Focus group discussion.

Though none of the included studies explicitly reported their findings as per the constructs of the acceptability framework (Panel 1); the themes generated from the analysis were mapped with these constructs. A summary of the constructs addressed by each of the included paper is shown in Table 5. The table immediately highlights ‘ethicality’ or ethical implications of the intervention and ‘opportunity cost’ of implementing or participating in the interventions were the least explored concepts in the included studies, particularly for women. All constructs of the framework were more likely to be explored if health workers, funders or staff from the Ministry of Health were the study participants. Affective attitude was reported in all papers including women.

**Table 5:** Constructs of the theoretical framework of acceptability explored in included papers by stakeholder types.

#### Affective atttitude

**Women and their families** were happy when they feel they have benefited directly from the intervention, especially when the intervention appears to have contributed to a good outcome.

*“We think that the child was saved by the nurses because of the timeliness of our arrival. We were happy when we got into the hands of the nurses.” (Woman, Ghana, Patel et al. N.D)*

Though women were generally happy to participate in various mobile/text-based interventions, some women reported getting confused or worried by some of the messages received while at home.

*“It is clear to me although I got confused when I received the message about what to do when the baby is not moving at all … I asked the nurse because that got me really scared but she managed to make me understand.” (Woman, South Africa, Skinner 2018)*

**Health workers** were happy participating in community interventions as it benefits women and creates avenue for capacity building for them.

*“I personally felt good because now I had something that I would be taking to them and teach them about IFAS* [Iron and folic acid supplementation] *in details. I felt that this was going to help many women.” (Female Community Health volunteer, Kenya, Kamau 2020)*

However, some health workers highlighted their displeasure at the increased workload as a result of the intervention and some unpleasant encounters with women and their families.

*“It complicates and increases our work. One has to stop everything she is doing and concentrate when sending reports. When you have a baby and family responsibilities it is difficult and then it creates conflict with family responsibilities.”(Community Health volunteer, Rwanda Mwendwa, 2015)*

*“Give us the money that you have been given instead of tormenting us with your questions.”(Community health workerrecounting field experience*, *Tanzania, Joseph, 2021)*

**Funders/staff from ministries of health**: externally funded intervention may appear to have ambitious targets to local stakeholders at the Ministry of Health and there were concerns about power dynamics in multi-partner consortium interventions affecting how these stakeholders feel about the intervention.

*“It was a very ambitious goal that in the first year [we would have a] 50% reduction in MMR* [maternal mortality ratio]. *We looked at people [SMGL partners] and said, “Are you going to make this? This goal is very high.” (host government, Palaia 2019)*

*“And by big P [Partner] and small p [partner], it had to do with who had the biggest investments and therefore gets the biggest seat at the table. So that was a little bit concerning for us, because those big P partners seemed to have had more of the say in the partnership.” (small partner in consortium, Palaia 2019)*

#### Burden and barriers

None of the included papers explored the burden or barriers faced by women who are the recipients of the intervention, but these were explored for health workers and other stakeholders.

**Health workers** implementing community interventions in some cases had to overcome geographical and sociocultural barriers to discharge their duties.

*“Rains were a hindrance to our efforts in sensitising the community on the use of ANC services. We used to hold public meetings in open spaces but when it rained, we had to postpone them.” (Community health worker, Tanzania, Joseph 2021)*

*“You know for me as a man, visiting peoples’ homes week after week might raise some questions somewhere.” (Male CHV, Kenya, Kamau, 2020)*

Health workers were also faced with challenges of insufficient training, lack of necessary equipment or tools to carry out intervention and in some cases, community health volunteers were not remunerated.

*“Equipment are not yet available, we need the right equipment to take measures from pregnant women and children. For example, we need instruments to measure the height and weight of pregnant women because those measures were not taken at the health facility. We also need thermometers to measure temperature.” (Community Health worker, Rwanda Musabyimana, 2018)*

*“My partner always wanted to know the amount of money I was paid. No matter how often I told him that we were not paid, he never understood. You know, when you spend such a long time walking all over the village and come back home very tired with nothing in your pocket, very few partners will understand why you keep committed to the intervention.” (Community health volunteer, Tanzania, Joseph 2021)*

**Local stakeholders and funders** felt the burden of implementation was eased by drawing on the unique strengths and experiences of consortium partners, however, they reported that strong leadership was required for consortia to function optimally.

*“I think the partnership was aiming to achieve first of all, having a pool of varied resources. So we have a lot more than if we had one or two people involved, both financial as well as the technical support and understanding. And also just bringing the varied experiences from the different partners, from the very beginning.” (Field representative Uganda or Zambia, Palaia 2019)*

*I think that effective leadership made a difference— there was always a sense of team. And that doesn’t happen without effort. There was remarkably little ego, which is really hard to do with these separate agencies with their own separate missions coming together for one mission as a team, so a lot of that was just really strong leadership and management. (Global Partner, Palaia 2019)*

#### Ethicality

The construct ethicality examined the extent to which interventions fit with an individual’s value system. None of the included papers explored ethicality of interventions for women, but it was explored for health workers and other stakeholders.

**For health workers**, interventions that involved trained community volunteers delivering some health services in the community led to internal conflicts about task delegation.

*“I would say the health professionals are much better, we cannot solely give the responsibility to the CHVs. We have to do this professionally, bearing in mind the woman is pregnant, there can be other problems.” (Nurse in charge of ANC services, Kenya, Kamau, 2020)*

A community health volunteer reported hiding information about the intervention from her spouse to enable her to continue engaging with the program. Health workers who reported lack of support from their spouses attributed it to the little or no remuneration received from project participation.

*“At times I failed to tell my husband where I was going. This is because he was not supportive of the interventions we were implementing. I knew the risk of telling him where I was going daily since I knew his reaction.” (Community health volunteer, Tanzania, Joseph 2021)*

Although intervention funders desire higher levels of host government engagement at all stages of an intervention, high profile officials were often unavailable at the initial stages of community intervention design and as such, interventions maybe designed without the level of leadership input desired.

*They [the MOH] weren’t even represented on the Leadership Council in the early days. And I think that was a serious mistake and something that I hope has been corrected and will continue to be corrected. The problem is that you don’t have high officials in a host government who are willing to sit through long conference calls or travel to Washington for meetings talking about leadership and governance. (US Government representative, Palaia 2019)*

There were concerns about rightful access and use of project data, as health workers felt that having access to the data could inform better service provision.

*“We don’t have access to the information in RapidSMS. The data manager and the CHW [community health workers] supervisor are the only ones who have access to this information. For example, we would like to be able to check whether all the women who delivered at our health center were registered in RapidSMS, because we have their name in our registers.”-Provider from a District not supported by UNICEF (Community health volunteer, Rwanda, Musabyimana, 2018)*

#### Intervention coherence

**Women and their families** showed an understanding of what each intervention programme offered and, in some cases, offered suggestions on how they could be improved.

*“Sometimes you get it in the morning, but usually you get it around 9:00 or 10:00 … Yes, but sometimes it delays and you receive it late … And sometimes when there is a delay, they often send a message apologising. (Woman, South Africa, Skinner 2018)*

*I wonderwhether pregnant women should provide their phone number since they are the beneficiaries. Then both she and the CHW* [community health worker] *would receive the RapidSMS messages. This system would help remind parents; we are very busy and we sometimes forget all our responsibilities to our children.” (Father in urban area, Rwanda,* Musabyimana *2018*)

**Health workers** showed a good understanding of the service delivery gaps targeted by the community interventions and of how the interventions work. They also proffered insights on how to maximise the benefits afforded by the interventions.

*“We worked closely with health workers, and in the facilities and this made our work persuasive. For instance, whenever we referred pregnant women to the facility for more information, health workers would attend to them very well. Similarly, whenever we invited them to attend our community sensitisation meetings, they would come and help us in clarifying some of the health issues.” (Community health volunteer, Tanzania, Joseph 2021)*

*“The system needs to point out mistakes, otherwise, we send the same wrong mistake many times”. (Community Health volunteer, Rwanda Mwendwa, 2015)*

**Partners** who fund community intervention projects try to engender local ownership by ensuring local stakeholders are involved at every stage and understand the intervention and how it works.

*“Partners have not done activities in the district without consulting the DHO [District Health Office], the Chief Administrative Officer, and with the Chief Administrative Office, the District Executive Committee. And monthly there have been project coordination meetings and that makes us own whatever we do, that we are implementing in these areas.” (Subnational Government representative Uganda, Palaia 2019)*

However, lack of clarity on partner roles can hinder progress in multi-stakeholder collaboration projects.

*“The partners were kind of cobbled together pretty quickly, it seemed without a lot of thought of what would they do, how they would contribute in distinctive ways. And that's something that took a long time to resolve, and I'm not sure it even really was resolved.” (Global Partner, Palaia 2019)*

#### Opportunity costs

**Women and their families** reported having to sometimes endure some discomfort to benefit from the intervention with the hopes of achieving the desired good outcomes for the mother and baby. In an intervention involving the use of make-shift motorbike ambulance which was considered uncomfortable compared to the traditional ambulance, a family member had this to say:

*“There are issues like discomfort, safety and others when you are being transported but as a sick person you do not have those issues in mind when there is an emergency. Anything that can hurriedly get you to the place on time is what you will be looking for. All vehicles have the tendency of falling when transporting people so it will not be fair relating safety issues to the Motorking alone.” (Male family member, Ghana, Patel et al. N.D)*

**Health workers** sometimes had to make sacrifices of time, money and logistics planning to make the interventions work.

*“I Often, I arrived home late because I had to walk a long distance from my home to the meeting venue. I would get back home late after meetings and I often found my little children asleep.” (Community health worker, Tanzania, Joseph 2021)*

*“In addition, the phones given to CHWs are old. Now they are buying phones using their own money. Providing them with new phones would provide them with a type of motivation.” (Community supervisor, Rwanda,* Musabyimana *2018)*

*“Most of the time ambulances are not available. We then have to use traditional transport to take the mother to the nearest health centre. Mothers at times deliver on the way to the health centre before an ambulance comes.” (Community Health volunteer, Rwanda Mwendwa, 2015)*

**Funders and staff of ministries of health**: When funding organizations had rigid timelines and difficult bureaucratic processes, host governments report having to forgo their desired level of groundwork to ensure that the funder timelines are met.

*“I think some of the groundwork that would ordinarily happen when trying to put together a partnership of this size; it just didn't because speed seemed really important. There's this real desire to get something off the ground quickly, and so there wasn’t the planning and the groundwork that you would usually see with something like this until it was catching up and learning more information, figuring out how to plug in.” (Host government representative, Palaia 2019)*

#### Perceived effectiveness

**Women and their families** generally believed that the interventions were helpful, and shared personal stories of positive difference made by the interventions.

*“They (MomConnect messages) are so helpful because even after you give birth, they also tell you how to take care of the baby; in case you notice something wrong with the baby, go to the clinic.” (Woman, South Africa, Skinner 2018).*

*“If not for the Motorking* [motorbike ambulance] *women especially pregnant women and children would have been suffering a lot …. It is able to go to the interior [of communities] to carry cases like the one I told you about with the woman who was in labour and nearly died if not for the sake of the Motorking ambulance.” (Community sub-chief, Ghana, Patel* et al. *N. D)*

**Health workers** and community volunteers believed that the interventions contribute to reduction in mortality, improved demand for antenatal care and that recipients were in turn cascading the benefits of the intervention to other women in their social circles.

*“I can see we dropped maternal mortality we reached Millennium Development Goals, about reducing maternal mortality and the impact is very positive for me. Maybe statistically you can’t report that is related to RapidSMS only because there were so many interventions in the area.” (Central level participant, Rwanda,* Musabyimana *2018*)

*“Antenatal care attendance improved because CHVs [*community health volunteers*] were really mobilizing mothers to come to the clinic. For the deliveries we had, I would say we did not have many serious cases due to low Hb. Also, IFAS [Iron and Folic Acid supplementation] adherence improved.” (Nurse in charge of ANC services, Kenya, Kamau, 2020)*

*“We have visited them during this program of IFAS* [Iron and folic acid supplementation]*and they have been taking them and now they are teaching the other women about the importance of taking IFAS and other services as well.” (Community health volunteer, Kenya, Kamau, 2020)*

**Funders and staff of ministries of health**: Funders consider a project to be effective if they secure host government buy-in and commitment to scale the intervention. In addition, the ability to work with multiple agencies and achieve useful outcome data was reported as a measure of project effectiveness.

*“The district and local leadership were very excited about it. And then when it started to show pretty incredible successes, the government really got behind it, embraced it and wanted to roll it up and package it as one of their everyday work”. (Host government representative, Uganda or Zambia, Palaia 2019)*

*“It’s kind of a hallmark of SMGL [saving mothers giving life] that we don't just produce fluff, we actually provide health outcome data, which is extremely rare in USAID-led projects. I’m very proud of the M&E [monitoring and evaluation] we have done and our ability to work across agency silos capturing outcomes in a really sterling, top-notch way.” (US Government representative, Palaia 2019)*

#### Self-efficacy

**Women** felt empowered to make the right decisions for their health and that of their babies due to the support they received from the interventions.

*“When I registered with MomConnect they informed me that ‘I must pack my ID, my clothes, sanitary towels and the child’s clothes’, things like that … It was simple to raise a child with MomConnect because I took every advice I got from MomConnect”. (Woman, South Africa, Skinner 2018)*

**Health workers** felt more capable of doing their jobs and some desired more training to maximise the use of available information.

*“There are many benefits because I learned that IFAS increases the blood levels in a woman and it makes her with baby to be healthy. It helps the mothers to avoid so many problems, which they face when giving birth. We learned the sources of iron and how to cook like spinach and not to overcook which we pass to the mothers and teach the same thus creating awareness to the community.” (Community Health volunteer, Kenya, Kamau 2020)*

*“What needs to be improved is the use of the data we are collecting. Health facility staff and everyone who has access to RapidSMS should be trained on data analysis so that they can benefit from the system and know what type of information is provided by the system****.”****(Community supervisor, Rwanda,* Musabyimana, *2018)*

**Local stakeholders** reported that the integration of the intervention infrastructures and resources with the government mechanisms increased their chances of sustaining the gains of donor-funded interventions.

*“A lot of infrastructure improvements have been done … and equipment—those can probably stay longer. Maybe, five years or more. A lot of capacity has been built of the health workers and a number of them have been taken on by the districts of the government of Uganda. They have been put on the government payroll, so I believe with that knowledge that has been passed on to them, that is something that can stay on in the long term.” (Uganda Government representative, Palaia 2019)*

## Discussion

This systematic review of community-based interventions for stillbirths assessed the types, completeness of reporting and acceptability of interventions in SSA since 2000. A total of 39 reports from 34 studies, conducted in 18 countries in SSA were included in this review. All four regions of SSA—west, east, central and southern Africa were represented in the study. The quality of included studies ranged from poor to good, with the majority receiving a fair rating. Four types of interventions were identified: nutritional, infection prevention and treatment, improving access to skilled childbirth attendants and improving women’s health knowledge and behaviours. These interventions were delivered using nine strategies including mHealth, women groups, community midwifery, home visits, mass media sensitisation, traditional birth attendants and community volunteer training, community mobilisation and transport vouchers. The least reported area for the included interventions were intervention tailoring, modification and intervention fidelity. Community-based interventions were generally acceptable to women, health workers and local ministries of health. However, important constructs of acceptability such as ethicality, opportunity cost and burden of intervention have been poorly explored for women in this region.

While there are unique contextual differences in LMICs there is room for adaptation of successful interventions from one context to the other. With many public health interventions being incompletely described in published literature,^55^ this potential benefit has not been fully exploited. Many interventions targeting stillbirths are often complex interventions; described as interventions which have several interacting components,^56^ it is important for researchers to describe each component in replicable details for future researchers to attain similar level of intervention efficacy. Similar to previous reviews, this review showed that details about intervention intensity, dosing, tailoring and modification are incompletely reported.^55^^,^^57^^,^^58^ Though it may appear counter-intuitive for researchers to report some intervention components such as intensity, dosing or tailoring for some study designs (e.g cross sectional studies), it may be beneficial for future researchers and implementers to know for instance, the frequency, variations or tailoring of training visits over time or across geographical zones within the study. Skivington and colleagues^8^ described a comprehensive framework for developing and evaluating complex interventions as one having core elements (including understanding context, engaging stakeholders in developing programme theory) and four phases—feasibility studies, intervention development, implementation and evaluation. They argue that researchers that study complex interventions need to address and report a broader range of questions including unintended impacts of research, how it interacts with the health system and how their findings can support future researchers or policy makers in making decisions. Incomplete reporting of interventions also poses additional challenges for researchers synthesising available evidence in a meta-analysis as some characteristics may remain uncertain, hampering effective moderator analysis.^59^

There is evidence that community-based interventions can complement interventions at the health facilities to accelerate progress for stillbirth reduction in SSA. Scaling up these interventions and instituting policies based on evidence generated from them will require both robust intervention documentation and assessments. Evidence synthesis from studies across the world suggest different level of effectiveness of different interventions implemented for stillbirth prevention at the various stages of pregnancy.^9^^,^^60^, ^61^, ^62^ Commonly recommended interventions before and during pregnancy include periconceptional folic acid supplementation, malaria screening and treatment, syphilis screening and treatment. As the pregnancy advances, other recommended interventions include detection and management of diabetes and hypertensive disorders of pregnancy, detection of fetal growth restriction and post-term pregnancy. During childbirth, interventions recommended are skilled childbirth attendance, basic and comprehensive emergency obstetric care. Only interventions outside of the clinical setting were examined in this review and the interventions were grouped into types based on what it sought to accomplish^9^ and the intervention strategies deployed.^10^ This review showed that various types of community-based interventions for preventing stillbirths have been tested in countries located in all four subregions of SSA, potentially due to increasing policy attention and collaboration for stillbirth (and broader maternal and newborn health) in this region.^5^^,^^6^

In SSA, 22% of childbirths occur in health facilities,^63^ and 64% have skilled childbirth attendance.^64^ Increasing access to skilled attendance at childbirth is one of the evidence-based recommendations for reducing stillbirths in SSA.^9^ This reviews shows that several strategies have been tried to improve skilled attendance at childbirth in the region with varying degrees of success. Strategies tried include emergency transport, health worker training, training traditional birth attendants (TBA), free maternal health services, community midwifery etc. Of these strategies, TBA training remains one of the most controversial with inconclusive findings on its effectiveness as some studies have found them to be beneficial in improving maternal and newborn outcome including reducing stillbirths,^65^^,^^66^ and others found them unhelpful.^31^ While there is a preference for skilled attendance at childbirth, the worsening shortages in human resources for health in SSA require innovative strategies to make up for the shortfall.^67^ Some researchers argue that TBAs could potentially serve to reduce the gaps in primary maternal care, if they are adequately trained, supervised and integrated into the health system. In Timor Leste TBAs have been incorporated into the national healthcare system through Family Health promoter programme since 2007 and has since been scaled up and assessed to have contributed in the reduction of poor maternal and newborn health outcomes in the country.^68^

Maternal infection with several viral and bacterial pathogens is thought to account for 20%–50% of stillbirths in low-middle income settings.^9^^,^^69^ Malaria and syphilis infection have been associated with a two-fold increase in the risk of stillbirths,^70^ while uncontrolled HIV has been associated with over 1.5-fold risk.^71^ This risk of stillbirth can be reduced at the community level through disease control and prevention efforts (e.g use of insecticide treated nets) or massive screening campaigns coupled with treatment; strategies which were both identified by this review. Several non-clinical interventions which aim to alter maternal nutrition and behaviours leading to improved maternal health status and reduction of fetal exposure to insult have been attempted with various degrees of success.^72^ Similar to previous reviews, researchers in the included studies adopted strategies such as quality antenatal education, women groups, home visits and community mobilisation to achieve this. Despite the concerns for the inequitable access to mobile health (mHealth) interventions due to inadequate mobile phone penetration in SSA (46% in 2020^73^), mHealth interventions for health education, appointment reminders and referral coordination are rapidly increasing in SSA,^7^ although many of them lack robust evaluations to identify their impact on health outcomes.^7^ A notable intervention included in this review is the rapid SMS intervention in Rwanda which was piloted as a mechanism to support community health workers delivering community maternal and newborn health interventions. This intervention has now been scaled nationally and is utilised for several health service delivery programmes.^74^

This review uncovered important issues about how the acceptability of interventions has been studied in SSA. Recommendations for community-based interventions advocate for co-creation, co-implementation and co-evaluation with host communities as acceptability by the potential beneficiaries is crucial for success and sustainability.^75^ Although intervention acceptability alone may not substantially influence the intervention outcomes, different studies have linked acceptability of interventions with increased adoption or engagement with intervention, compliance or adherence, user satisfaction and experience, effectiveness or impact and sustainability beyond implementation phase.^76^, ^77^, ^78^ The findings highlight that various aspects of the interventions could be related to acceptability, including affective attitude, intervention burden and barriers, ethicality, intervention coherence, opportunity costs, perceived effectiveness and self-efficacy attained from interventions targeting women and their families. Health workers, women and their families expressed happiness to partake in community interventions due to the perceived direct benefits from participation. However, similar to another study, health workers also highlighted challenges such as increased workload, conflicts with family responsibilities, the need for better support and remuneration.^79^ Ethical considerations were examined, particularly from the perspective of health workers and other stakeholders, but not for women and their families which could be a major problem in improving acceptability of community-based interventions. Task delegation and conflicts regarding the involvement of community volunteers were highlighted. Although task-shifting policy is increasingly being adopted to address the wide human resource gaps in many SSA countries, its implementation is not without challenges including inadequate training, inadequate supportive supervision, lack of clarity on roles, ethical concerns for health outcome implications and legal protection for additional tasks ceded.^80^ It was interesting to find that despite having no remuneration some community volunteers continued to support interventions, aligning with previous findings that volunteers are mostly driven by the intrinsic desire to serve, and gain satisfaction and status within the community.^76^^,^^81^ Women and health workers demonstrated a good understanding of their roles in intervention activities, but as reported in previous studies, navigating bureaucracies in multi-partner consortium of funders and the local ministries of health can be complicated, inadvertently influencing the outcome of the intervention. Funders and staff from ministries of health had concerns about the ambitious targets set by externally funded interventions, emphasising the importance of strong local leadership and engagement with potential beneficiary communities to ensure the success of interventions.

A lot of attention on intervention acceptability in the included studies were channelled towards the health care workers implementing these interventions rather than women, probably because of the protracted critical workforce shortages in the region.^67^ Interventions in the region need to be driven by local ministries of health to improve sustainability. The Rapid SMS program in Rwanda, included in this review, is a government-led mHealth intervention which started in 2010 and from various assessments, has been found to significantly contribute to improved outcomes such as decreased maternal and infant mortality, and increased demand for high quality maternal and newborn health services.^74^ An important finding of this review was that none of the qualitative evaluations addressed the ethical implications and burden of the interventions from the perspectives of the women and their families. This brings the participation of women under scrutiny as one may wonder if they participate in these interventions by choice or out of despondency. There is need to increase engagement of women in co-creation, co-implementation and co-evaluation of stillbirth prevention interventions trials. Recently, Kim et al.,^82^ reported a minimum set of key outcomes (core outcome set) agreed on by stakeholders that should be reported consistently in all efficacy and effectiveness trials for stillbirth prevention. While this appears to be a useful development for stillbirth prevention, Dube et al.,^83^ argue that the involvement of parents and families from high burden LMICs for the outcome set development appear ‘tokenistic’ and therefore may not reflect the needs of these parents or communities. The perspectives and experiences of women and their families can inform contextually appropriate strategies that address the unique challenges and cultural considerations of these settings, leading to improved outcomes for both women and babies.

Regarding the strengths and limitations, this is an updated review of community-based interventions for stillbirth prevention in SSA and to the best of our knowledge provides the first assessment of acceptability and completeness of reporting of stillbirth prevention interventions in SSA. This review needs to be interpreted bearing the following limitations in mind. First, only recent interventions documented and published in peer-reviewed journals and grey literature from 2000 to date were included. It is possible that some community interventions may have been documented in paper records or news briefs and hence could not be included in this review. Secondary data from seven studies were utilised for the assessment of intervention acceptability and this is not a comprehensive review of acceptability of community-based interventions, hence the inferences from the findings may be limited; however, this does not diminish the importance of findings especially relating to acceptability for women.

There are many types of community-based interventions implemented through various strategies for stillbirth prevention in sub-Saharan Africa. The benefits of these interventions are undermined by the incomplete reporting hence limiting the replicability, intervention scale-up and reliability of the robustness of their evaluation. There is need for periodic holistic evaluation of intervention including their acceptability to health actors, particularly women who are the primary potential beneficiaries.

## Contributors

UGA, MN, CO and JJK conceptualised this review. NR was responsible for library resources and supervision of the resource retrieval from databases. NR and UGA conducted the relevant searches. UGA, YYB, and AO were responsible for data collection and analysis. CO, JJK, MN and NR were responsible for validation and supervision. UGA prepared the initial draft of the paper, and all authors contributed to the development and refinement of subsequent drafts. UGA, YYB and AO mined and have verified the underlying data for this study. UGA, YYB, AO, MN, CO, NR, JJK read and approved the final manuscript for submission. UGA, YYB, AO, NR, JJK and CO had access to the data in the study and accept responsibility for the decision to submit for publication.

## Data sharing statement

All the data relevant to this study have been included in the article or included as supplementary information.

## Declaration of interests

All authors declare no competing interests.
