# Supplementary material for: Types, reporting and acceptability of community-based interventions for stillbirth prevention in sub-Saharan Africa (SSA): a systematic review
Source: eClinicalMedicine. 2023 Aug 3;62:102133. doi: 10.1016/j.eclinm.2023.102133 (PMC10430180; doi:10.1016/j.eclinm.2023.102133)
Supplement: Supplementary materials [file mmc1.pdf]

Supplementary Material

Table of Contents

Appendix 1: Detailed search strategy 2

Appendix 2: Quality assessment of included studies.. 9

Appendix 3: Tidier Checklist assessment of included studies.. 12

Appendix 4: Acceptability constructs and supporting quotes from included studies.. 14

Appendix 1: Detailed search strategy

CINAHL(EBSCOhost)[1982-present]	
#	Query
S19	S4 AND S12 AND S15 AND S18
S18	S16 OR S17
S17	TX Angola or Benin or Botswana or "Burkina Faso" or Burundi or Cameroon or "Cape Verde" or "Cabo verde" or "Central African Republic" or Chad or Comoros or Congo or Djibouti or "Equatorial Guinea" or Eritrea or Ethiopia or Gabon or Gambia or Ghana or Guinea or "Ivory Coast" or "Cote d'Ivoire" or Jamahiriya or Jamahiryia or Kenya or Lesotho or Liberia or Libya or Libia or Madagascar or Malawi or Mali or Mauritania or Mauritius or Mayote or Mozambique or Mocambique or Namibia or Niger or Nigeria or Principe or Reunion or Rwanda or "Sao Tome" or Senegal or Seychelles or "Sierra Leone" or Somalia or "South Africa" or "St Helena" or Sudan or Swaziland or Tanzania or Togo or Tunisia or Uganda or "Western Sahara" or Zaire or Zambia or Zimbabwe or "Central Africa" or "Central African" or "West Africa" or "West African" or "Western Africa" or "Western African" or "East Africa" or "East African" or "Eastern Africa" or "Eastern African" or "South African" or "Southern Africa" or "Southern African" or "sub Saharan Africa" or "sub Saharan African" or "subSaharan Africa" or "subSaharan African"
S16	(MH "Africa") OR (MH "Africa South of the Sahara+")
S15	S13 OR S14
S14	TI ((stillbirth* or ((perinatal or fetal or foetal or fetus or intrauterine or intra-uterine) N1 (death* or mortality)))) OR AB ((stillbirth* or ((perinatal or fetal or foetal or fetus or intrauterine or intra-uterine) N1 (death* or mortality))))
S13	(MH "Perinatal Death") OR (MH "Pregnancy Complications/MO") OR (MH "Pregnancy Outcomes")
S12	S5 OR S6 OR S7 OR S8 OR S9 OR S10 OR S11
S11	TI (((community N2 intervention*) or community mobilization or community mobilisation or birth attendant* or community health worker* or community worker* or home visit* or women* groups or mother* groups or mobile health or mhealth or m-health or mobile phone* or cellphone* or cell phone* or smartphone* or health promotion or health education or national program* or nutritional intervention* or nutritional program* or smoking cessation or ((stop* or quit*) N2 smok*))) OR AB (((community N2 intervention*) or community mobilization or community mobilisation or birth attendant* or community health worker* or community worker* or home visit* or women* groups or mother* groups or mobile health or mhealth or m-health or mobile phone* or cellphone* or cell phone* or smartphone* or health promotion or health education or national program* or nutritional intervention* or nutritional program* or smoking cessation or ((stop* or quit*) N2 smok*)))
S10	(MH "Cellular Phone+")
S9	(MH "Smoking Cessation") OR (MH "Smoking Cessation Programs")
S8	(MH "National Health Programs")
S7	(MH "Health Education") OR (MH "Nutrition Education") OR (MH "Health Promotion")
S6	(MH "Midwives+") OR (MH "Community Health Workers")
S5	(MH "Community Health Services") OR (MH "Community Programs") OR (MH "Community Health Centers")
S4	S1 OR S2 OR S3
S3	TI (pregnan* or maternal care) OR AB (pregnan* or maternal care)

S2	(MH "Maternal Health Services+")
S1	(MH "Pregnancy") OR (MH "Expectant Mothers")

**Web of Science Core Collection – Science Citation Index and Social Science Citation index [1900-present]**	
#	Query
1	TS=(pregnan* or maternal care)
2	TS=((community NEAR/2 intervention*) or community mobilization or community mobilisation or birth attendant* or community health worker* or community worker* or home visit* or women* groups or mother* groups or mobile health or mhealth or m-health or mobile phone* or cellphone* or cell phone* or smartphone* or health promotion or health education or national program* or nutritional intervention* or nutritional program* or smoking cessation or ((stop* or quit*) NEAR/2 smok*))
3	TS=(stillbirth* or ((perinatal or fetal or foetal or fetus or intrauterine or intra-uterine) NEXT (death* or mortality)))
4	TS=(Angola or Benin or Botswana or "Burkina Faso" or Burundi or Cameroon or "Cape Verde" or "Cabo verde" or "Central African Republic" or Chad or Comoros or Congo or Djibouti or "Equatorial Guinea" or Eritrea or Ethiopia or Gabon or Gambia or Ghana or Guinea or "Ivory Coast" or "Cote d'Ivoire" or Jamahiriya or Jamahiryia or Kenya or Lesotho or Liberia or Libya or Libia or Madagascar or Malawi or Mali or Mauritania or Mauritius or Mayote or Mozambique or Mocambique or Namibia or Niger or Nigeria or Principe or Reunion or Rwanda or "Sao Tome" or Senegal or Seychelles or "Sierra Leone" or Somalia or "South Africa" or "St Helena" or Sudan or Swaziland or Tanzania or Togo or Tunisia or Uganda or "Western Sahara" or Zaire or Zambia or Zimbabwe or "Central Africa" or "Central African" or "West Africa" or "West African" or "Western Africa" or "Western African" or "East Africa" or "East African" or "Eastern Africa" or "Eastern African" or "South African" or "Southern Africa" or "Southern African" or "sub Saharan Africa" or "sub Saharan African" or "subSaharan Africa" or "subSaharan African") OR CU=(Angola or Benin or Botswana or "Burkina Faso" or Burundi or Cameroon or "Cape Verde" or "Cabo verde" or "Central African Republic" or Chad or Comoros or Congo or Djibouti or "Equatorial Guinea" or Eritrea or Ethiopia or Gabon or Gambia or Ghana or Guinea or "Ivory Coast" or "Cote d'Ivoire" or Jamahiriya or Jamahiryia or Kenya or Lesotho or Liberia or Libya or Libia or Madagascar or Malawi or Mali or Mauritania or Mauritius or Mayote or Mozambique or Mocambique or Namibia or Niger or Nigeria or Principe or Reunion or Rwanda or "Sao Tome" or Senegal or Seychelles or "Sierra Leone" or Somalia or "South Africa" or "St Helena" or Sudan or Swaziland or Tanzania or Togo or Tunisia or Uganda or "Western Sahara" or Zaire or Zambia or Zimbabwe or "Central Africa" or "Central African" or "West Africa" or "West African" or "Western Africa" or "Western African" or "East Africa" or "East African" or "Eastern Africa" or "Eastern African" or "South African" or "Southern Africa" or "Southern African" or "sub Saharan Africa" or "sub Saharan African" or "subSaharan Africa" or "subSaharan African")
5	#1 AND #2 AND #3 AND #4

**Medline (Ovid MEDLINE® Epub Ahead of Print, In-Process & Other Non-Indexed Citations, Ovid MEDLINE® Daily and Ovid MEDLINE®) 1946 to present**	
#	Query
1	Pregnancy/ or Pregnant Women/
2	exp Maternal Health Services/
3	(pregnan* or maternal care).mp.
4	1 or 2 or 3
5	community health services/ or community participation/
6	Midwifery/ or Community Health Workers
7	health education/ or health promotion/
8	National Health Programs/

9	Smoking Cessation/
10	exp Cell Phone/
11	((community adj2 (intervention* or program*)) or community mobilization or community mobilisation or midwives or midwifery or birth attendant* or community health worker* or community worker* or home visit* or women* groups or mother* groups or mobile health or mhealth or m-health or mobile phone* or cellphone* or cell phone* or smartphone* or text messag* or health promotion or health education or national program* or nutrition* intervention* or nutrition* program* or smoking cessation or ((stop* or quit*) adj2 smok*)).mp.
12	5 or 6 or 7 or 8 or 9 or 10 or 11
13	perinatal death/ or fetal death/ or stillbirth/
14	fetal mortality/ or perinatal mortality/
15	Pregnancy Outcome/
16	Pregnancy Complications/mo
17	(stillbirth* or ((perinatal or fetal or foetal or fetus or intrauterine or intra-uterine) adj (death? or mortality))).mp.
18	13 or 14 or 15 or 16 or 17
19	Africa/ or exp "Africa South of the Sahara"/
20	(Angola or Benin or Botswana or "Burkina Faso" or Burundi or Cameroon or "Cape Verde" or "Cabo verde" or "Central African Republic" or Chad or Comoros or Congo or Djibouti or "Equatorial Guinea" or Eritrea or Ethiopia or Gabon or Gambia or Ghana or Guinea or "Ivory Coast" or "Cote d'Ivoire" or Jamahiriya or Jamahiryia or Kenya or Lesotho or Liberia or Libya or Libia or Madagascar or Malawi or Mali or Mauritania or Mauritius or Mayote or Mozambique or Mocambique or Namibia or Niger or Nigeria or Principe or Reunion or Rwanda or "Sao Tome" or Senegal or Seychelles or "Sierra Leone" or Somalia or "South Africa" or "St Helena" or Sudan or Swaziland or Tanzania or Togo or Tunisia or Uganda or "Western Sahara" or Zaire or Zambia or Zimbabwe or "Central Africa" or "Central African" or "West Africa" or "West African" or "Western Africa" or "Western African" or "East Africa" or "East African" or "Eastern Africa" or "Eastern African" or "South African" or "Southern Africa" or "Southern African" or "sub Saharan Africa" or "sub Saharan African" or "subSaharan Africa" or "subSaharan African").mp.
21	19 or 20
22	4 and 12 and 18 and 21

WHO Global Index Medicus – limit to AIM (**globalindexmedicus.net)**	
#	Query
1	(pregnan* OR maternal) AND (community OR "birth attendant*" OR "home visit*" OR "womens groups" OR "mothers groups" OR "mobile health" OR mhealth OR m-health OR "mobile phone*" OR cellphone* OR "cell phone*" OR smartphone* OR "health promotion" OR "health education" OR "national program*" OR "nutritional intervention*" OR "nutrition intervention*" OR "nutrition program*" OR "nutritional program*"<br>OR "smoking cessation" OR "stop smoking" OR "quit smoking") AND (stillbirth* OR "perinatal death*" OR "perinatal mortality" OR "fetal death*" OR "fetal mortality" OR "foetal death*" OR "foetal mortality" OR "fetus death*" OR "fetus mortality" OR "foetus death*" OR "foetus mortality" OR "intrauterine death*" OR "intrauterine mortality" OR "intra-uterine death*" OR "intra-uterine mortality")

**Global Health 1973 to present**	
#	Query
1	Pregnancy/ or Pregnant Women/
2	(pregnan* or maternal care).mp.
3	1 or 2

4	community health/ or community health services/ or community involvement/ or community nutrition/ or community programmes/
5	community health workers/ or midwives/ or traditional birth attendants/
6	health education/ or health promotion/
7	health programmes/
8	Smoking Cessation/
9	mobile telephones/
10	nutrition programmes/ or nutritional interventions/
11	((community adj2 (intervention* or program*)) or community mobilization or community mobilisation or midwives or midwifery or birth attendant* or community health worker* or community worker* or home visit* or women* groups or mother* groups or mobile health or mhealth or m-health or mobile phone* or cellphone* or cell phone* or smartphone* or text messag* or health promotion or health education or national program* or nutrition* intervention* or nutrition* program* or smoking cessation or ((stop* or quit*) adj2 smok*)).mp.
12	4 or 5 or 6 or 7 or 8 or 9 or 11
13	fetal death/ or stillbirths/
14	perinatal mortality/
15	(stillbirth* or ((perinatal or fetal or foetal or fetus or intrauterine or intra-uterine) adj (death? or mortality))).mp.
16	13 or 14 or 15
17	exp "africa south of sahara"/
18	(Angola or Benin or Botswana or "Burkina Faso" or Burundi or Cameroon or "Cape Verde" or "Cabo verde" or "Central African Republic" or Chad or Comoros or Congo or Djibouti or "Equatorial Guinea" or Eritrea or Ethiopia or Gabon or Gambia or Ghana or Guinea or "Ivory Coast" or "Cote d'Ivoire" or Jamahiriya or Jamahiryia or Kenya or Lesotho or Liberia or Libya or Libia or Madagascar or Malawi or Mali or Mauritania or Mauritius or Mayote or Mozambique or Mocambique or Namibia or Niger or Nigeria or Principe or Reunion or Rwanda or "Sao Tome" or Senegal or Seychelles or "Sierra Leone" or Somalia or "South Africa" or "St Helena" or Sudan or Swaziland or Tanzania or Togo or Tunisia or Uganda or "Western Sahara" or Zaire or Zambia or Zimbabwe or "Central Africa" or "Central African" or "West Africa" or "West African" or "Western Africa" or "Western African" or "East Africa" or "East African" or "Eastern Africa" or "Eastern African" or "South African" or "Southern Africa" or "Southern African" or "sub Saharan Africa" or "sub Saharan African" or "subSaharan Africa" or "subSaharan African").mp.
19	Africa/ or exp "Africa South of the Sahara"/
20	17 or 18
21	3 and 12 and 16 and 19

**Embase 1974 to present**	
#	Query
1	Pregnancy/ or Pregnant Woman/
2	Maternal Health Service/
3	(pregnan* or maternal care).mp.
4	1 or 2 or 3
5	community care/ or community program/ or community participation/
6	midwife/ or traditional birth attendant/ or health auxiliary/
7	health education/ or health promotion/
8	health program/
9	Smoking Cessation/
10	exp mobile phone/
11	((community adj2 intervention*) or community mobilization or community mobilisation or birth attendant* or community health worker* or community worker* or home visit* or women* groups

	or mother* groups or mobile health or mhealth or m-health or mobile phone* or cellphone* or cell phone* or smartphone* or health promotion or health education or national program* or nutritional intervention* or nutritional program* or smoking cessation or ((stop* or quit*) adj2 smok*)).mp.
12	5 or 6 or 7 or 8 or 9 or 10 or 11
13	exp fetus death/ or perinatal death/
14	fetus mortality/ or exp perinatal mortality/ or prenatal mortality/
15	*Pregnancy Outcome/
16	(stillbirth* or ((perinatal or fetal or foetal or fetus or intrauterine or intra-uterine) adj (death? or mortality))).mp.
17	13 or 14 or 15 or 16
18	exp "africa south of the sahara"/ or africa/
19	(Angola or Benin or Botswana or "Burkina Faso" or Burundi or Cameroon or "Cape Verde" or "Cabo verde" or "Central African Republic" or Chad or Comoros or Congo or Djibouti or "Equatorial Guinea" or Eritrea or Ethiopia or Gabon or Gambia or Ghana or Guinea or "Ivory Coast" or "Cote d'Ivoire" or Jamahiriya or Jamahiryia or Kenya or Lesotho or Liberia or Libya or Libia or Madagascar or Malawi or Mali or Mauritania or Mauritius or Mayote or Mozambique or Mocambique or Namibia or Niger or Nigeria or Principe or Reunion or Rwanda or "Sao Tome" or Senegal or Seychelles or "Sierra Leone" or Somalia or "South Africa" or "St Helena" or Sudan or Swaziland or Tanzania or Togo or Tunisia or Uganda or "Western Sahara" or Zaire or Zambia or Zimbabwe or "Central Africa" or "Central African" or "West Africa" or "West African" or "Western Africa" or "Western African" or "East Africa" or "East African" or "Eastern Africa" or "Eastern African" or "South African" or "Southern Africa" or "Southern African" or "sub Saharan Africa" or "sub Saharan African" or "subSaharan Africa" or "subSaharan African").mp.
20	18 or 19
21	4 and 12 and 17 and 20

**ProQuest Dissertations & Theses Global (proquest.com)**	
#	Query
1	noft((pregnan* or "maternal care")) AND noft(((community NEAR2 intervention*) or community mobilization or community mobilisation or birth attendant* or community health worker* or community worker* or home visit* or women* groups or mother* groups or mobile health or mhealth or m-health or mobile phone* or cellphone* or cell phone* or smartphone* or health promotion or health education or national program* or nutritional intervention* or nutritional program* or smoking cessation or ((stop* or quit*) NEAR2 smok*))) AND noft((stillbirth* or ((perinatal or fetal or foetal or fetus or intrauterine or intra-uterine) NEAR2 (death* or mortality))))

**Cochrane Central Register of Controlled Trials(Cochrane Library, Wiley)[1ssue 7 of 12, July 2022]**	
#	Query
1	MeSH descriptor: [Pregnancy] this term only
2	MeSH descriptor: [Pregnant Women] explode all trees
3	MeSH descriptor: [Maternal Health Services] explode all trees
4	#1 OR #2 OR #3
5	MeSH descriptor: [Community Health Services] this term only
6	MeSH descriptor: [Community Participation] this term only
7	MeSH descriptor: [Community Health Workers] explode all trees
8	MeSH descriptor: [Midwifery] explode all trees
9	MeSH descriptor: [Health Promotion] this term only
10	MeSH descriptor: [Health Education] this term only
11	MeSH descriptor: [National Health Programs] this term only
12	MeSH descriptor: [Smoking Cessation] explode all trees
13	MeSH descriptor: [Cell Phone] explode all trees

14	(((community NEAR/2 intervention*) or community mobilization or community mobilisation or birth attendant* or community health worker* or community worker* or home visit* or women* groups or mother* groups or mobile health or mhealth or m-health or mobile phone* or cellphone* or cell phone* or smartphone* or health promotion or health education or national program* or nutritional intervention* or nutritional program* or smoking cessation or ((stop* or quit*) NEAR/2 smok*))):ti,ab,kw
15	#5 OR #6 OR #7 OR #8 OR #9 OR #10 OR #11 OR #12 OR #13 OR #14
16	MeSH descriptor: [Perinatal Death] explode all trees
17	MeSH descriptor: [Fetal Death] explode all trees
18	MeSH descriptor: [Perinatal Mortality] explode all trees
19	MeSH descriptor: [Fetal Mortality] explode all trees
20	((stillbirth* or ((perinatal or fetal or foetal or fetus or intrauterine or intra-uterine) NEXT (death* or mortality)))):ti,ab,kw
21	#16 OR #17 OR #18 OR #19 OR #20
22	#4 AND #15 AND #21
23	MeSH descriptor: [Africa South of the Sahara] explode all trees
24	MeSH descriptor: [Africa] this term only
25	(Angola or Benin or Botswana or "Burkina Faso" or Burundi or Cameroon or "Cape Verde" or "Cabo verde" or "Central African Republic" or Chad or Comoros or Congo or Djibouti or "Equatorial Guinea" or Eritrea or Ethiopia or Gabon or Gambia or Ghana or Guinea or "Ivory Coast" or "Cote d'Ivoire" or Jamahiriya or Jamahiryia or Kenya or Lesotho or Liberia or Libya or Libia or Madagascar or Malawi or Mali or Mauritania or Mauritius or Mayote or Mozambique or Mocambique or Namibia or Niger or Nigeria or Principe or Reunion or Rwanda or "Sao Tome" or Senegal or Seychelles or "Sierra Leone" or Somalia or "South Africa" or "St Helena" or Sudan or Swaziland or Tanzania or Togo or Tunisia or Uganda or "Western Sahara" or Zaire or Zambia or Zimbabwe or "Central Africa" or "Central African" or "West Africa" or "West African" or "Western Africa" or "Western African" or "East Africa" or "East African" or "Eastern Africa" or "Eastern African" or "South African" or "Southern Africa" or "Southern African" or "sub Saharan Africa" or "sub Saharan African" or "subSaharan Africa" or "subSaharan African"):ti,ab,kw
26	#24 OR #25
27	#22 AND #26

**WHO International Clinical Trials Registry**	https://trialsearch.who.int/
#	Query
1	Pregnancy OR Pregnant (title row) AND Community(intervention row)
2	(pregnant OR pregnancy) AND community AND (stillbirth OR stillbirths OR fetal death OR fetal deaths OR fetal mortality OR perinatal death OR perinatal deaths OR perinatal mortality)

**ClinicalTrials**	https://www.clinicaltrials.gov/
#	Query
1	Stillbirth OR Perinatal death OR Perinatal mortality OR fetal death OR fetal mortality(title row) AND Community(intervention row)
2	Pregnancy OR Pregnant(Condition row) AND Community (intervention row)
3	(pregnant OR pregnancy) AND community AND (stillbirth OR stillbirths OR fetal death OR fetal deaths OR fetal mortality OR perinatal death OR perinatal deaths OR perinatal mortality) (Other terms)

**Google**

#	Query
1	(pregnancy OR pregnant) AND (community OR "birth attendants" OR "home visits" OR "womens groups" OR "mothers groups" OR "mobile health" OR mhealth OR m-health OR "mobile phones" OR cellphones OR "cell phones" OR smartphones OR "health promotion" OR "health education" OR "nutritional interventions" OR "nutrition interventions" OR programs OR programmes OR "smoking cessation" OR "stop smoking" OR "quit smoking") AND (stillbirths OR "perinatal deaths" OR "perinatal mortality" OR "fetal deaths" OR "fetal mortality" OR "foetal deaths" OR "foetal mortality" OR "fetus deaths" OR "fetus mortality" OR "foetus deaths" OR "foetus mortality" OR "intrauterine deaths" OR "intrauterine mortality" OR "intra-uterine deaths" OR "intrauterine mortality")
2	(pregnancy OR pregnant) AND (community OR "birth attendants" OR "home visits" OR mhealth OR "mobile phones" OR "health education" OR "nutritional interventions" OR programs OR "smoking cessation" AND (stillbirths OR "perinatal deaths") site:.org

Appendix 2: Quality assessment of included studies

Quality assessment for Pre-post studies - National Heart, Lung, and Blood Institute Appraisal Checklist													
S/N	First author and year	1	2	3	4	5	6	7	8	9	11	12	Total
1	Sloan 2018	1	1	1	1	1	0	1	0	1	1	1	8(75)
2	Seim 2014	1	1	1	1	1	0	1	0	0	0	0	6(50)
3	Serbanescu 2019	1	1	1	0	1	0	1	0	0	0	0	5(45)
4	Sibley 2014	1	1	1	0	1	1	1	0	1	1	N/A	8(73)
5	Conlon 2019	1	1	1	0	1	0	1	0	0	0	0	5(45)
6	Hounton 2009	1	1	0	0	1	0	1	0	1	0	0	5(45)
7	Shikuku 2020	1	0	0	0	0	1	1	0	1	0	0	5(45)
8	Okonofua 2023	1	1	1	1	1	1	1	0	N/A	0	N/A	7(78)

1. Was the research question or objective in this paper clearly stated?
2. Were eligibility/selection criteria for the study population prespecified and clearly described?
3. Were the participants in the study representative of those who would be eligible for the test/service/intervention in the general or clinical population of interest?
4. Were all eligible participants that met the prespecified entry criteria enrolled?
5. Was the sample size sufficiently large to provide confidence in the findings?
6. Was the test/service/intervention clearly described and delivered consistently across the study population?
7. Were the outcome measures prespecified, clearly defined, valid, reliable, and assessed consistently across all study participants?
8. Were the people assessing the outcomes blinded to the participants' exposures/interventions?
9. Did the statistical methods examine changes in outcome measures from before to after the intervention? Were statistical tests done that provided p values for the pre-to-post changes?
11. Were outcome measures of interest taken multiple times before the intervention and multiple times after the intervention (i.e., did they use an interrupted time-series design)?
12. If the intervention was conducted at a group level (e.g., a whole hospital, a community, etc.) did the statistical analysis take into account the use of individual-level data to determine effects at the group level?

1: fully reported; 0: Not reported; N/A: Not applicable

Quality assessment for qualitative studies - Critical Appraisal Checklist												
S/N	First author and year	1	2	3	4	5	6	7	8	9	10	Total (%)
1	Palaia 2019	1	1	0	1	0	0	1	0	1	1	6(60)
2	Joseph 2021	1	1	1	0	1	0	1	0	1	1	7(70)
3	Kamau 2020	1	1	1	1	1	0	1	1	1	1	9(90)
4	Skinner 2018	1	1	1	0	1	0	0	1	1	1	7(70)
5	Musabyimana 2018	1	1	1	1	1	0	1	1	1	1	9(90)
6	Mwendwa 2015	1	1	1	1	1	0	1	0	1	1	8(80)

7	Patel et al N.D	1	0	1	1	1	0	1	0	1	1	8(80)
8	Azaare 2022	1	1	1	0	1	0	1	0	1	1	7(70)
	1. Was there a clear statement of the aims of the research?<br>2. Is a qualitative methodology appropriate?"<br>3. Was the research design appropriate to address the aims of the research?<br>4. Was the recruitment strategy appropriate to the aims of the research?<br>5. Was the data collected in a way that addressed the research issue?<br>6. Has the relationship between researcher and participants been adequately considered?<br>7. Have ethical issues been taken into consideration?<br>8. Was the data analysis sufficiently rigorous?<br>9. Is there a clear statement of findings?<br>10. How valuable is the research?<br><br>1: fully reported/shown; 0: Not reported; N/A: Not applicable											

**Quality assessment for cross-sectional and cohort studies - National Heart, Lung, and Blood Institute**															
S/N	**First author and year**	**1**	**2**	**3**	**4**	**5**	**6**	**7**	**8**	**9**	**10**	**11**	**12**	**13**	**14**	**Total (%)**
1	Fatti 2016	1	1	0	1	0	1	1	0	1	0	1	0	0	1	8(57)
2	Okafor 2015	1	1	1	1	0	1	1	0	0	0	1	0	N/A	0	7(54)
3	Headstrom 2022	1	1	1	1	0	1	1	0	1	1	1	0	1	1	11(79)
4	Mostert 2021	1	1	0	1	1	1	1	0	1	0	1	0	0	1	9(64)
5	Makuluni 2021	1	1	0	1	0	1	1	0	0	0	1	0	0	0	6(43)
	1. Was the research question or objective in this paper clearly stated?<br>2. Was the study population clearly specified and defined?<br>3. Was the participation rate of eligible persons at least 50%?<br>4. Were all the subjects selected or recruited from the same or similar populations (including the same time period)? Were inclusion and exclusion criteria for being in the study prespecified and applied uniformly to all participants?<br>5. Was a sample size justification, power description, or variance and effect estimates provided?<br>6. For the analyses in this paper, were the exposure(s) of interest measured prior to the outcome(s) being measured?<br>7. Was the timeframe sufficient so that one could reasonably expect to see an association between exposure and outcome if it existed?<br>8. For exposures that can vary in amount or level, did the study examine different levels of the exposure as related to the outcome (e.g., categories of exposure, or exposure measured as continuous variable)?<br>9. Were the exposure measures (independent variables) clearly defined, valid, reliable, and implemented consistently across all study participants?<br>10. Was the exposure(s) assessed more than once over time?<br>11. Were the outcome measures (dependent variables) clearly defined, valid, reliable, and implemented consistently across all study participants?<br>12. Were the outcome assessors blinded to the exposure status of participants?<br>13. Was loss to follow-up after baseline 20% or less?<br>14. Were key potential confounding variables measured and adjusted statistically for their impact on the relationship between exposure(s) and outcome(s)?1: fully reported; 0: Not reported; N/A: Not applicable															

Quality assessment for (cluster) Randomised control trials –Cochrane Risk of Bias II assessment tool								
**S/N**	**First author and year**	**1a**	**1b**	**2**	**3**	**4**	**5**	**6**
1	Mohammed 2016	High	Some concerns	Low	Low	Low	Low	Some concerns
2	Colbourn 2013	Low	Low	Low	Low	Low	Low	Low
3	Lewycka 2013	Some concerns	Low	Low	Low	High	Low	Some concerns
4	Lund 2014	Some concerns	Low	Low	Low	Low	Low	Some concerns
5	Alexander 2018	Low	Low	Low	Low	Low	Low	Low
6	Matendo 2011	Some concerns	Low	Low	Low	Low	Low	Some concerns
7	Leight 2018	High	High	Low	Low	Low	Some concerns	High
8	Scott 2019	Low	Low	Low	Low	Low	Low	Low
9	Pasha 2013	Some concerns	Low	Low	Low	Low	Low	Some concerns
10	Ilboudo et al 2022	Low	Some concerns	Low	Low	Low	Low	Some concerns
11	Kone et al 2022	Low	Low	Low	Low	Low	Low	Low
12	De Kok et al 2022	Low	Low	Low	Low	Low	Low	Low
		Domain 1a. Randomization process<br>Domain 1b: Risk of bias arising from the timing of identification or recruitment of participants<br>Domain 2. Deviations from intended interventions<br>Domain 3. Missing outcome data<br>Domain 4. Measurement of the outcome<br>Domain 5. Selection of the reported result<br>Domain 6. Overall Bias						

Appendix 3: Tidier Checklist assessment for included studies

		Tidier assessment criteria								
S/N	**First author and year**	**1**	**2**	**3**	**4**	**5**	**6**	**7**	**8**	**9**
1	Mohammed 2016	Y	Y	Y	Y	Y	Y	NR	NR	NR
2	Chen 2011	Y	Y	Y	Y	Y	Y	Y	Y	NR
3	Colbourn 2013	Y	Y	Y	Y	Y	Y	Y	Y	NR
4	Gloyd 2001	Y	Y	Y	Y	Y	Y	NR	NR	NR
5	Kaestel 2005	Y	Y	Y	Y	Y	Y	NR	NR	NR
6	Lewycka 2011	Y	Y	Y	Y	Y	Y	NR	NR	NR
7	Lund 2014	Y	Y	Y	Y	Y	Y	NR	NR	NR
8	Okafor 2015	Y	Y	Y	Y	NR	NR	NR	NR	NR
9	Seim 2014	Y	Y	Y	Y	Y	Y	NR	Y	NR
10	Serbanescu 2019	Y	Y	Y	Y	Y	NR	Y	Y	NR
11	Sibley 2014	Y	Y	Y	Y	Y	Y	Y	Y	Y
12	Pasha 2013	Y	Y	Y	Y	Y	Y	Y	Y	Y
13	Hounton 2009	Y	Y	Y	Y	Y	Y	NR	NR	NR
14	Alexander 2018	Y	Y	Y	Y	Y	Y	NR	Y	NR
15	Fatti 2016	Y	Y	Y	Y	Y	Y	NR	Y	NR
16	Joseph 2021	Y	Y	Y	Y	Y	NR	NR	NR	NR
17	Kamau 2020	Y	Y	Y	Y	Y	Y	NR	NR	NR
18	Matendo 2011	Y	Y	Y	Y	Y	Y	NR	NR	NR
19	Mostert 2021	Y	Y	Y	Y	Y	NR	NR	NR	NR
20	Skinner 2018	Y	Y	Y	Y	Y	NR	NR	NR	NR
21	Sloan 2018	Y	Y	Y	Y	Y	Y	NR	NR	NR
22	Scott 2019	Y	Y	Y	Y	Y	NR	NR	NR	NR
23	Shikuku 2020	Y	Y	Y	Y	Y	Y	NR	NR	NR
24	Leight 2018	Y	Y	Y	Y	Y	Y	NR	NR	NR
25	Musabyimana 2018	Y	Y	Y	Y	Y	NR	NR	NR	NR
26	Bryan 2017	Y	Y	Y	Y	Y	NR	NR	NR	NR
27	Patel N.D	Y	Y	Y	Y	Y	Y	NR	NR	NR
28	Makuluni & Stones	Y	Y	NR	NR	NR	NR	NR	NR	NR
29	Azaare et al 2022	Y	Y	Y	Y	Y	Y	NR	NR	NR
30	Ilboudo et al 2022	Y	Y	Y	Y	Y	Y	Y	Y	NR
31	Kone et al 2022	Y	Y	Y	Y	Y	Y	Y	Y	Y
32	de Kok et al., 2022	Y	Y	Y	Y	Y	Y	Y	Y	Y
33	Headstrom et al., 2022	Y	Y	Y	Y	Y	Y	Y	Y	Y
34	Okonofua et al., 2023	Y	Y	Y	Y	Y	Y	NR	NR	NR

1. Why? Described any rationale, theory or goal of the elements essential to the intervention
2. What? Materials – describe physical or informational materials used in the intervention. Procedure: describe each of the procedures, activities and/or processes used in the intervention including enabling or support activities
3. Who provided? For each category of intervention provider, describe their expertise, background and any specific training given
4. How? Describe the modes of intervention delivery (eg face to face or by some other mechanism such as the internet or telephone). Was it delivered individually or in a group?
5. Where? Describe the types of location where the intervention occurred, including any necessary infrastructure or relevant features
6. When and how much? Describe the number of times the intervention was delivered and over what period of time including the number of sessions, their schedule, and their duration, intensity or dose
7. Tailoring. If the intervention was planned to be personalised titrated or adapted, then describe what, why, when and how

	8. Modification.If the intervention was modified during the course of the study, describe the changes (what, why, when, and how).<br>9. How well? Planned: If intervention adherence or fidelity was assessed, describe how and by whom, and if any strategies were used to maintain or improve fidelity, describe them.<br>Actual: if intervention adherence or fidelity was assessed, describe the extent to which the intervention was delivered as planned.<br>Y: fully reported, NR: Not reported

Appendix 4: Acceptability constructs and supporting quotes from included studies

First author and year	Acceptability construct and supporting quotes in included papers
Palaia 2019	**1. Affective Attitude (how an individual feels about the intervention)**<br>*It was a very ambitious goal that in the first year [we would have a] 50% reduction in MMR. We looked at people [SMGL partners] and said, "Are you going to make this? This goal is very high." And they said, "It is good to aim high and then see how things work. At least we got to 30%. And then progressively we'll be able to reduce the maternal mortality ratio by more than 30%." (subnational host government)*<br><br>*I know that there's the typical sort of bureaucratic challenges, rivalries, funding challenges—all the things that are always inherent in any kind a project. It just seemed like [the partners] really had the mission first and foremost in mind ... I think that's one of things that made SMGL function, was that the partners were sort of aligned on the key topline objective. (goal alignment - global partner)*<br><br>*Huge decisions like how many years to keep SMGL going [were] largely driven by funding. ... I think the USG held a huge role in decision making because we had the big purse. (power dynamics – big global partner)*<br>*[A]nd by big P [Partner] and small p [partner], it had to do with who had the biggest investments and therefore gets the biggest seat at the table. So that was a little bit concerning for us, because those big P partners seemed to have had more of the say in the partnership (small global partner)*<br><br>**2. Burden (perceived amount of effort that is required to participate in the intervention)**<br>*I think the partnership was aiming to achieve first of all, having a pool of varied resources. So we have a lot more than if we had one or two people involved, both financial as well as the technical support and understanding. And also just bringing the varied experiences from the different partners, I think, from the very beginning (partner expertise – field representative)*<br><br>*I think that effective leadership made a difference— there was always a sense of team. And that doesn't happen without effort. There was remarkably little ego, which is really hard to do with these separate agencies with their own separate missions coming together for one mission as a team, so a lot of that was just really strong leadership and management, and tone setting, those types of things. (effective leadership - global leader)*<br><br>**3. Ethicality (the extent to which the intervention has good fit with an individual's value system)**<br>*They [the MOH] weren't even represented on the Leadership Council in the early days. And I think that was a serious mistake and something that I hope has been corrected and will continue to be corrected. The problem is that you don't have high officials in a host government who are willing to sit through long conference calls or travel to Washington for meetings talking about leadership and governance (US govt rep – govt representation in programs)*<br><br>**4. Intervention Coherence (the extent to which the participant understands the intervention and how it works)**

	The partners were kind of cobbled together pretty quickly, it seemed without a lot of thought of what would they do, how they would contribute in distinctive ways. And that's something that took a long time to resolve, and I'm not sure it even really was resolved (partnership role clarity – global partner)<br><br>*Partners have not done activities in the district without consulting the DHO [District Health Office], the Chief Administrative Officer, and with the Chief Administrative Office, the District Executive Committee. And monthly there have been project coordination meetings and that makes us own whatever we do, that we are implementing in these areas. (Country ownership - sub national govt)*<br><br>**5. Opportunity Costs (the extent to which benefits, profits or values must be given up to engage in the intervention)**<br>*I think some of the ground work that would ordinarily happen when trying to put together a partnership of this size, it just didn't because speed seemed really important. There's this real desire to get something off the ground quickly, and so there wasn't the planning and the groundwork that you would usually see with something like this until it was catching up and learning more information, figuring out how to plug in. So it wasn't the ideal dynamic (local partner)*<br>*I think in any partnership, a funding cycle has different sorts of decision makers and timelines, and I would not say we were fast. There were several delays in our funding, but it was often [because], you know, we didn't have congressional approval or things like that. So it's hard to control but it's the reality of how funding gets allocated. (Global partner – bureaucracy)*<br><br>**6. Perceived Effectiveness (the extent to which the intervention is perceived as likely to achieve its purpose)**<br>*It's kind of a hallmark of SMGL that we don't just produce fluff, we actually provide health outcome data, which is extremely rare in USAID-led projects. I'm very proud of the M&E [monitoring and evaluation] we have done and our ability to work across agency silos capturing outcomes in a really sterling, top-notch way. (strength of MEL – US govt rep)*<br><br>*The district and local leadership were very excited about it. And then when it started to show pretty incredible successes, the government really got behind it, embraced it and wanted to roll it up and package it as one of their everyday work. (Country ownership - sub national govt)*<br><br>**7. Self efficacy (the Participant's confidence that they can perform the behaviour(s) required to participate in the intervention**<br>*A lot of infrastructure improvements have been done ... and equipment—those can probably stay longer. Maybe, five years or more. A lot of capacity has been built of the health workers and a number of them have been taken on by the districts of the government of Uganda. They have been put on the government payroll, so I believe with that knowledge that has been passed on to them, that is something that can stay on in the long term (sustainability – country national government)*
Joseph 2021	**1. Affective Attitude (how an individual feels about the intervention)**<br><br>*Make your story very short; I have other businesses to attend to. After all, what you are saying is not new to me.*

	I am old enough to know my responsibility when pregnant, you are still too young to advise me. (WG in MDC recounts embarrassments from clients)<br><br>*If anything bad happens to me you will be liable.*<br>*I will deal with whoever told you that I am pregnant.*<br>*Give us the money that you are given instead of tormenting us with your questions.*<br>*(WG in KDC recounts embarrassments from clients)*<br><br>**2. Burden (perceived amount of effort that is required to participate in the intervention)**<br>*Rains were a hindrance to our efforts in sensitising the community on the use of ANC services. We used to hold public meetings in open spaces but when it rained, we had to postpone them. Attendance by community members in these meetings during the peak of the rainy season was also poor. In several occasions, we postponed the meetings in the middle as rains continued. In some months, it rained consecutively for the whole week and thus disrupted our work plan (FGD with WGS, in MDC).*<br><br>*My partner always wanted to know the amount of money I was paid. No matter how often I told him that we were not paid, he never understood. You know, when you spend such a long time walking all over the village and come back home very tired with nothing in the pocket, very few partners will understand why you keep committed to the intervention* (IDI with WG, in MDC).<br><br>**3. Ethicality (the extent to which the intervention has good fit with an individual's value system)**<br>*.At times I failed to tell my husband where I was going. This is because he was not supportive of the interventions we were implementing. I knew the risk of telling him where I was going daily since I knew his reaction" (IDI with WG, in MDC).*<br><br>**4. Intervention Coherence (the extent to which the participant understands the intervention and how it works)**<br>*We worked closely with health workers; and in the facilities and this made our work persuasive. For instance, whenever we referred pregnant women to the facility for more information, health workers would attend to them very well. Similarly, whenever we invited them to attend our community sensitisation meetings, they would come and help us in clarifying some of the health issues (IDI with WGS in KDC – Health system support).*<br><br>*We are the one who received clients who were sensitized by the WGs. And in most cases, we used to attend sensitisation meetings that were held at the village level and clarified several ANC issues that were technical such family planning issues (IDI with WG Supervisor in KDC).*<br><br>**5. Opportunity Costs (the extent to which benefits, profits or values must be given up to engage in the intervention)**<br>*I Often, I arrived home late because I had to walk a long distance from my home to the meeting venue. I would get back home late after meetings and I often found my little children asleep. This most often annoyed my husband (IDI with WG, in MDC).*
Kamau 2020	**1. Affective Attitude (how an individual feels about the intervention)**

	"She was very happy to see us at her home because if she did not take IFAS in order to increase her blood levels and improve on her pregnancy maybe there were going to be some complications. . .. . . she explained to me how she felt after giving birth that she had enough energy and did not have low blood levels." (Female CHV, health facility 2)<br><br>*"I personally felt good because now I had something that I would be taking to them and teach them about IFAS in details. I felt that this was going to help many women" (Female CHV, health facility 5).*<br>*"This was a good project because it bridged a gap and an area {IFAS counselling} which health workers have not taken seriously." (Nurse in charge of ANC services, health facility 1)*<br><br>*"This one for the community approach is good because they must bring IFAS for you, and they emphasize on the importance. You see that they consider it important and you also become serious" (ANC woman, health facility 2)*<br><br>**2. Burden (perceived amount of effort that is required to participate in the intervention)**<br>*"I can say dealing with pregnant women is not easy since they tend to have bad moods and I would find one in a bad mood who would not even want us to talk and she thinks the IFAS are for my own benefit and not hers" (Female CHV, health facility 3)*<br><br>*"Just a little bit, you know for me as a man, visiting peoples' home week after week might raise some questions somewhere. But now considering that I have been doing other jobs at the grass roots level it was not a huge challenge because I had explained well to them why I was following up on IFAS for pregnant women" (Male CHV, health facility 1)*<br><br>*. ..at times you call someone, the husband picks the phone, you talk for some time trying to explain who you are to them because that is someone's else wife you called. . .." (Male CHV, health facility 2)*<br><br>*"It was not tedious on our side. When the client is supposed to take IFAS from CHV, you spend less time with them." (Nurse in charge of ANC services, health facility 2)*<br><br>*"The major problem is lack of facilitation {remuneration}." (Male CHV, health facility 1)*<br><br>*". . .. . .sometimes he did not come on time and kept me waiting" (ANC woman, health facility 1, IDI)*<br><br>**3. Ethicality (the extent to which the intervention has good fit with an individual's value system)**<br>*"I would say the health professionals are much better, we cannot solely give the responsibility to the CHVs. We have to do this professionally, bearing in mind the woman is pregnant. . . . . .there can be other problems." (Nurse in charge of ANC services, health facility 2)*<br><br>**4. Intervention Coherence (the extent to which the participant understands the intervention and how it works)**

	"In short, taking IFAS from the hospital or facility should not be the end but there should be ways of following up especially to the grass roots because I have realized that some of the mothers who get IFAS from the hospital throw them in the toilets or they just keep and do not take them" (Male CHV, health facility 1)<br><br>**6. Perceived Effectiveness (the extent to which the intervention is perceived as likely to achieve its purpose)**<br>*"The number of clients who have come to the facility has increased because we are able now to reach them, to reach more clients so that they can come to the facility and . . .." (Female CHV, health facility 3)*<br><br>*"Aaaah, they are the ones who were looking for you to tell you that so and so is pregnant, and they need to begin ANC early. . .. . ." (Female CHV, health facility 3)*<br><br>*". . .we have visited them during this program of IFAS and they have been taking them and now they are teaching the other women about the importance of taking IFAS . . .. .and other services as well" (Female CHV, health facility 3)*<br><br>*antenatal care attendance improved because CHVs were really mobilizing mothers to come to the clinic. For the deliveries we had, I would say we did not have many serious cases due to low Hb. Also, IFAS adherence improved." (Nurse in charge of ANC services, health facility 1)*<br><br>*"The ones who are far will access the supplements easily . . .. I come from far, so it is easy for them to bring me the supplements and the transport has reduced" (ANC woman, health facility 2)*<br><br>**7. Self efficacy (the Participant's confidence that they can perform the behaviour(s) required to participate in the intervention**<br>*"There are many benefits because I learned that IFAS increases the blood levels in a woman and it makes her with baby to be healthy. It helps the mothers to avoid so many problems which they face when giving birth. We learned the sources of iron and how to cook like spinach and not to overcook which we pass to the mothers and teach the same thus creating awareness to the community" (Female CHV, health facility 2).*<br><br>*"When I came for the first time, I was taught on how to give IFAS to mothers in the community . . .. .we started the job immediately and when we started we were able to get a lot of pregnant women in our villages and we taught them on how to use IFAS. . .. . .." (Male CHV, health facility 2)*
Skinner 2018	**1. Affective Attitude (how an individual feels about the intervention)**<br><br>*I'd like to encourage (the) MomConnect program, it needs to go further because it will help many more people. (KZN M)*<br>*It is clear to me although I got confused when I received the message about what to do when the baby is not moving at all...I asked the nurse because that got me really scared but she managed to make me understand. (FS P)*<br><br>**2. Burden (perceived amount of effort that is required to participate in the intervention)**

	Xhosa has deep words that are difficult to understand...Yes English at least you can even look up (English) in your dictionary. (WC M)<br><br>*No, it was just when I connected I accidentally put in Afri-kaans but then I reconnected again in English...Both mes-sages still come onto my phone but I only read the English ones because I don't understand Afrikaans. (GP M)*<br><br>**3. Ethicality (the extent to which the intervention has good fit with an individual's value system)**<br>*So I even showed a friend of mine who is pregnant and told her that she can dial this number because she is not connected on MomConnect...So I showed her how to do it from the paper. (WC P)*<br><br>*He can see that the message is written MomConnect, and when he reads it. For my side, if I left my phone at home, when I get back my partner tells me that I have a message from MomConnect. (FS P)*<br><br>**4. Intervention Coherence (the extent to which the participant understands the intervention and how it works)**<br>*Sometimes you get it in the morning, but usually you get it around 9:00 or 10:00...Yes, but sometimes it delays and you receive it late...And sometimes when there is a delay, they often send a message apologizing. (KZN M)*<br><br>**6. Perceived Effectiveness (the extent to which the intervention is perceived as likely to achieve its purpose)**<br>*They (MomConnect messages) are so helpful because even after you give birth, they also tell you how to take care of the baby; in case you notice something wrong with the baby, go to the clinic. (KZN M)*<br><br>*I do not want to lie I don't read posters, I don't get inter-ested in reading and learning about what is in the posters. (WC P)*<br><br>*They sent me something that my unborn has eye-brows, that way I can see my baby's development. (KZN P)*<br>*There was a time when my child couldn't pass the stools for twodays. I was at work and I called them. They told me that because she is still being breastfed. (MP M)*<br><br>**7. Self efficacy (the Participant's confidence that they can perform the behaviour(s) required to participate in the intervention**<br>*When I registered with MomConnect they informed me that 'I must pack my ID, my clothes, sanitary towels and the child's clothes' things like that...It was simple to raise a child with MomConnect because I took every advice I got from MomConnect. (MP P)*<br>*The messages play a huge role; like, you receive a message explaining something that you are already experiencing and you were about to go to the clinic 'for no reason'. (KZN M)*
Musabyimana 2018	**1. Affective Attitude (how an individual feels about the intervention)**

"They accept it! Now you will find women who take the initiative to look for a CHW to share the information that she is pregnant. Some also ask the CHW if the information about the fact that she is pregnant was sent through RapidSMS! After delivery, some also remind the CHW to send a message about the event. But this is not yet 100% of women."-CHW supervisor at HC level in a District not supported by UNICEF

"CHWs are very pleased to use the system! They are happy to see how they are assisting women and women are asking them to send both common and alert messages for their emergency cases."—CHW supervisor at HC level in a UNICEF supported District

2. Burden (perceived amount of effort that is required to participate in the intervention)
"*We are motivated to use the system. However, some community members have a poor mind-set regarding RapidSMS thinking that we are selling their information and getting paid for the information we send. As a result, sometimes they hide the needed information."—CHW from a District not supported by UNICEF*

"We were trained only once. The challenge we have is that we work with colleagues who were never trained".—CHW from a District not supported by UNICEF

"CHWs take measurements of the child. . .Okay! They have the tools, they take the measurements, then they classify children as "red" or "yellow" .But the indicators used confuse them. They mix them up: some use mid-upper arm circumference(MUAC),others weight for age, weight for height,. . .there is a need to have a look on how to train them better or maybe there is a need to recruit more qualified personnel. There is a need for improvement."—District official participant from a UNICEF supported District

"Equipment are not yet available.. .We need the right equipment to take measures from pregnant women and children. For example, we need instruments to measure the height and weight of pregnant women because those measures were not taken at the health facility.. ..We also need thermometers to measure temperature."—CHW from a non-supported Distric

"We know that we are volunteers and RapidSMS necessitates a lot of community-based work. It seems like there should be a financial motivation for all this work, but we do not receive it.. . .If we were paid for our time, we would be more motivated to work as CHWs because there would be compensation to help support our families."—CHW from a District not supported by UNICEF

3. Ethicality (the extent to which the intervention has good fit with an individual's value system)
"*We don't have access to the information in RapidSMS. The data manager and the CHW supervisor are the only ones who have access to this information. For example, we would like to be able to check whether all the women who delivered at our health center were registered in RapidSMS, because we have their name in our registers."-Provider from a District not supported by UNICEF*

4. Intervention Coherence (the extent to which the participant understands the intervention and how it works)
I wonder whether pregnant women should provide their phone number, since they are the beneficiaries. Then both she and the CHW would receive the RapidSMS messages.. . .This sys-tem would help remind parents; we are very busy and we sometimes forget all our responsibilities to our children".-

	Father from an urban area<br><br>*This would help someone living a long distance from the health center, as they could use RapidSMS to ensure they have an appointment. Healthcare providers could also then use it to see the order of consultations for the day. This would reduce the time patients spend queuing for an appointment."—Central level participant*<br><br>**5. Opportunity Costs (the extent to which benefits, profits or values must be given up to engage in the intervention)**<br>*"In addition, the phones given to CHWs are old. Now they are buying phones using their own money. Providing them with new phones would provide them with a type of motivation."—CHW supervisor at HC level in a UNICEF supported District*<br><br>**6. Perceived Effectiveness (the extent to which the intervention is perceived as likely to achieve its purpose)**<br>*"I can see we dropped maternal mortality we reached MDG, about maternal reducing maternal mortality we reached MDG.. .and the impact is very positive for me and may be statistically you can't report what is related to RapidSMS only because there were so many interventions in the area. But generally it is contributing so much toward the reduction of maternal land newborn and child mortality."—Central level participant*<br><br>*RapidSMS has helped a lot to prevent maternal, child and neonatal death. Through the collaboration between the health facility and CHWs and the information shared with RapidSMS, once the mother is reminded and attends the needed service on time, provider are motivated to do their best to keep the newborn and the mother alive".—CHW from a district not supported by UNICEF*<br><br>*". . .through RapidSMS I know when there is a newborn with this congenital abnormality and what health center they use. When this happens ,we can initiate contact with the hospital and physicians who perform the repair procedures for such children.. . .I get this information and can act without teven leaving my office."-CHW Supervisor from a district not supported by UNICEF*<br><br>*In our Monday staff meetings, we share information from the RapidSMS system with departments that can use it. For example, we give department-related information to the head of maternity care unit at the health center. The same is done for immunization services, and soon.. ."—Health center staff in a UNICEF-supported district*<br><br>**7. Self efficacy (the Participant's confidence that they can perform the behaviour(s) required to participate in the intervention**<br>*"What needs to be improved is the use of the data we are collecting. Health facility staff and everyone who has access to RapidSMS should be trained on data analysis so that they can benefit from the system and know what type of information is provided by the system."—CHW supervisor at DH from a District not supported by UNICEF*
Mwendwa 2015	**1. Affective Attitude (how an individual feels about the intervention)**

	We do not have to walk to the health centre to send a report. Mothers can directly be reported in the case of a problem.<br>*Nurses recognise our work, they know we work hard.*<br>*"If you send a message and it is confirmed you feel happy and feel encouraged to keep using RapidSMS''*<br><br>**2. Burden (perceived amount of effort that is required to participate in the intervention)**<br>*"We use our own knowledge because training is not enough"*<br><br>*"Most of our phones are old they don't work well"*<br><br>*''Sometimes there is an emergency and no battery charge then we cannot send RapidSMS. Again if you don't have the 100 Rwandan francs and the health centre is far, where do you charge?''*<br><br>*''RapidSMS is a new technology and is difficult sometimes. One has to consult the form with codes all the time and it takes our time.''*<br><br>*'It complicates and increases our work. One has to stop everything she is doing and concentrate when sending reports. When you have a baby and family responsibilities it is difficult and then it creates conflict with family responsibilities. Husbands are obstacles; they say we are supposed to be in the farm not reporting on RapidSMS.''*<br><br>*We have not used a technology before so it takes long to learn how to send the messages.*<br><br>*The codes are abbreviated in English; codes should be abbreviated in Kinyarawanda.*<br><br>*Sometimes we lose connectivity then we can't send SMS.*<br><br>**4. Intervention Coherence (the extent to which the participant understands the intervention and how it works)**<br>*The system needs to point out mistakes, otherwise we send the same wrong mistake many times.*<br><br>*Automated responses in emergency situations should include advice to CHWs on how to deal with the emergency.*<br><br>**5. Opportunity Costs (the extent to which benefits, profits or values must be given up to engage in the intervention)**<br>*''Most of the time ambulances are not available. We then have to use traditional transport to take the mother to the nearest health centre. Mothers at times deliver on the way to the health centre before an ambulance comes.''*<br><br>**6. Perceived Effectiveness (the extent to which the intervention is perveived as likely to achieve its purpose)**<br>*'Supervisors can know when one is having problems sending RapidSMS. They see in the system, who is sending reports, they help us during our monthly meeting.*

	RapidSMS helps us to send messages quickly and MOH gets reports immediately. Before we used papers which were stored at the health centre<br><br>*This kind of reporting makes the 'whole country' know a mother who is experiencing a difficulty and help can be sent to her"*<br><br>**7. Self efficacy (the Participant's confidence that they can perform the behaviour(s) required to participate in the intervention**<br>*Mothers now give us the information we need because they know we are communicating with the MOH.*<br>*''Supervisors can know when one is having problems sending RapidSMS. They see in the system, who is sending reports, they help us during our monthly meeting.''*
Patel N.D	**1. Affective Attitude (how an individual feels about the intervention)**<br><br>*"It sent me to the clinic to deliver and I did that safely without any bad thing happening to me. I delivered safely. That is the beauty of it."*<br>*"It has been so beneficial to the pregnant women and the children under-5 because they do not pay when the vehicle is transporting them. In the past, we used to transport pregnant women in donkey carts and on bicycles but today there is ready and reliable means of transport for them in emergencies." (Community volunteer during FGD)*<br>*"Everyone has his problem and when the vehicle picked me the driver knew that it was a painful thing being in labor so they also became careful with the way they were driving and we got there safely. Now I will not be able to speak for another person but for my experience it was comfortable." (IDI with female user of SERC services)*<br>*"We think that the child was saved by the nurses because of the timeliness of our arrival. We were happy when we got into the hands of the nurses." (IDI with female user of the service)*<br><br>**2. Burden (perceived amount of effort that is required to participate in the intervention)**<br>*"Our youth, if they could help us to repair our roads small, small and when the motors come, they can be running without problems."*<br><br>*"There are beasts at night and also ghosts. From where I come, there are so many spirits that it is not advisable to move out at night. The people sit protected in the vehicle whiles you are left alone in front. In addition to that you are not supposed to speed the vehicle and you can imagine how exposed you are if someone intends to harm you." (Driver during an FGD)*<br><br>**4. Intervention Coherence (the extent to which the participant understands the intervention and how it works)**<br>*"Some of the pregnant women will be complaining that they came and they are thrown away, they don't care about them. Because there is no understanding between the pregnant women and the midwife when she tells them it's not time for them to deliver and they should wait. Because of that, the women say the workers are not serious, but for me, the way I know about the work I know they are serious."*<br><br>**5. Opportunity Costs (the extent to which benefits, profits or values must be given up to engage in the intervention)**

	"There are issues like discomfort, safety and others when you are being transported but as a sick person you do not have those issues in mind when there is an emergency. Anything can hurriedly get you to the place on time is what you will be looking for. All vehicles have the tendency of falling when transporting people so it will not be fair relating safety issues to the Motorking alone." (Male FGD participant)that<br><br>**6. Perceived Effectiveness (the extent to which the intervention is perveived as likely to achieve its purpose)**<br>*If not for the Motorking, women especially pregnant women and children would have been suffering a lot.... It is able to go tothe interior [of communities] to carry cases like the one I told you about with the woman who was in labor and nearly died if not for the sake of the Motorking ambulance." (IDI with community sub-chief)*<br><br>*"When a woman is in labor and is not quickly sent to the health facility she might deliver. She might also lose either the baby or even herself. Kids like this, once they are weak, they can easily pass on. So the impact I see is that the emergency referral saves lives." (SERC driver during FGD)*
